# Enrichment of B cell receptor signaling and epidermal growth factor receptor pathways in monoclonal gammopathy of undetermined significance: a genome-wide genetic interaction study

**DOI:** 10.1186/s10020-018-0031-8

**Published:** 2018-06-11

**Authors:** Subhayan Chattopadhyay, Hauke Thomsen, Miguel Inacio da Silva Filho, Niels Weinhold, Per Hoffmann, Markus M. Nöthen, Arendt Marina, Karl-Heinz Jöckel, Börge Schmidt, Sonali Pechlivanis, Christian Langer, Hartmut Goldschmidt, Kari Hemminki, Asta Försti

**Affiliations:** 1 Division of Molecular Genetic Epidemiology, German Cancer Research Center (DKFZ), Im Neuenheimer Feld 580, 69120 Heidelberg, Germany; 2 Faculty of Medicine, University of Heidelberg, Heidelberg, Germany; 3 Department of Internal Medicine V, University of Heidelberg, Heidelberg, Germany; 4 Myeloma Institute, University of Arkansas for Medical Sciences, Little Rock, AR USA; 5 Institute of Human Genetics, University of Bonn, Bonn, Germany; 6 Department of Biomedicine, University of Basel, Basel, Switzerland; 7 Department of Genomics, Life & Brain Research Center, University of Bonn, Bonn, Germany; 8 Institute for Medical Informatics, Biometry and Epidemiology, University Hospital Essen, University of Duisburg-Essen, Essen, Germany; 9 Department of Internal Medicine III, University of Ulm, Ulm, Germany; 10 National Centre of Tumor Diseases, Heidelberg, Germany; 11 Center for Primary Health Care Research, Lund University, Malmö, Sweden

**Keywords:** MGUS, MM, Genome-wide interaction, Pathway, Network, B-cell signaling, EGFR signaling

## Abstract

**Background:**

Recent identification of 10 germline variants predisposing to monoclonal gammopathy of undetermined significance (MGUS) explicates genetic dependency of this asymptomatic precursor condition with multiple myeloma (MM). Yet much of genetic burden as well as functional links remain unexplained. We propose a workflow to expand the search for susceptibility loci with genome-wide interaction and for subsequent identification of genetic clusters and pathways.

**Methods:**

Polygenic interaction analysis on 243 cases/1285 controls identified 14 paired risk loci belonging to unique chromosomal bands which were then replicated in two independent sets (case only study, 82 individuals; case/control study 236 cases/ 2484 controls). Further investigation on gene-set enrichment, regulatory pathway and genetic network was carried out with stand-alone in silico tools separately for both interaction and genome-wide association study-detected risk loci.

**Results:**

Intronic-*PREX1* (20q13.13), a reported locus predisposing to MM was confirmed to have contribution to excess MGUS risk in interaction with *SETBP1*, a well-established candidate predisposing to myeloid malignancies. Pathway enrichment showed B cell receptor signaling pathway (*P* < 5.3 × 10^− 3^) downstream to allograft rejection pathway (*P* < 5.6 × 10^− 4^) and autoimmune thyroid disease pathway (*P* < 9.3 × 10^− 4^) as well as epidermal growth factor receptor regulation pathway (*P* < 2.4 × 10^− 2^) to be differentially regulated. Oncogene *ALK* and *CDH2* were also identified to be moderately interacting with rs10251201 and rs16966921, two previously reported risk loci for MGUS.

**Conclusions:**

We described novel pathways and variants potentially causal for MGUS. The methodology thus proposed to facilitate our search streamlines risk locus-based interaction, genetic network and pathway enrichment analyses.

**Electronic supplementary material:**

The online version of this article (10.1186/s10020-018-0031-8) contains supplementary material, which is available to authorized users.

## Background

Monoclonal gammopathy of undetermined significance (MGUS) is a premalignant phase of multiple myeloma (MM) and the most common plasma cell dyscrasia present in as high as 3.2% of general population below 50 years of age and up to 5.3% for population aged 70 years or older (Kyle et al. 2018). At an approximate annual rate of 1% MGUS progresses to MM, lymphoplasmacytic lymphoma/ Waldenström macroglobulinemia or amyloid light chain amyloidosis (AL amyloidosis) (Kyle et al. 2006; Dispenzieri et al. 2010). Apart from the knowledge of familial clustering from population studies, there has not been much development in deciphering the genetic architecture of MGUS (Greenberg et al. 2012; Frank et al. 2016; Landgren et al. 2009). Of late, 17 risk loci have been found predisposing to MM among which 9 are supposed to share association with MGUS (Mitchell et al. 2016; Thomsen et al. 2017; Weinhold et al. 2014; Greenberg et al. 2013).

A GWAS from our group on 243 German individuals with MGUS has recently discovered 10 susceptibility loci with varying degree of significance (Thomsen et al. 2017). GWAS on a genetic heterogeneous disorder such as MGUS would possibly be subject to ‘missing heritability’ which asserts, an association study can account for merely a small proportion of true causal genetic variations due to low detection power (Manolio et al. 2009). Linear interactions on paired single nucleotide polymorphisms (SNPs) can thus be used to inflate genomic resolution of test space and find novel risk locus pairs which otherwise would remain undetected. In a case/control approach statistical interaction models explain extra additive effects due to co-occurrences of two variants on top of the fixed effects (Cordell 2009). We use an unbiased statistic with high convergence rate, and observe increased detection power for genome-wide interacting pairs (Wellek and Ziegler 2009). Gene-set enrichment along with genome-wide pathway analysis that measures association of a phenotype to a predefined genetic cluster has become rather common in extending biological understanding of differentially regulated pathways affecting quantitative traits and phenotype variation (Ramanan et al. 2012; Khatri et al. 2012). This approach of mapping cancer susceptibility regions and detection of novel pathways in site-specific cancers has opened opportunities to new therapeutic approaches (Yi et al. 2017; Lee and Gyu Song 2015). Here we report a case/control interaction study on a discovery population of 243 MGUS cases and 1285 controls. The findings are subsequently supported by a case-only interaction study on 82 cases and finally confirmed with case/control interaction analysis on another population of 236 cases and 2484 controls. We pursue pathway enrichment analysis on a subsequent stage, based on both our current interaction study and the previous GWAS. To this end we use three in silico tools to discover novel pathways, corresponding genetic loci and clusters predisposing to MGUS taking into account significance of previously detected risk loci.

## Methods

### Ethics

Collection of samples and clinicopathological information from subjects was undertaken with the relevant ethical board approvals in accordance with the tenets of the Declaration of Helsinki. All subjects provided written informed consent. Ethical approval was obtained from Ethics committee of medicine faculty, University of Heidelberg, Heidelberg, Germany.

### Datasets

The University Clinic of Heidelberg and the University Clinic Ulm discovered 243 MGUS cases among which 114 (47%) were males with a mean age at diagnosis of 62 years, SD ± 11 years. The Ig isotype distribution was 72% IgG, 12% IgA, and 16% other Ig isotypes (Thomsen et al. 2017; Weinhold et al. 2014). These MGUS cases were identified during diagnostic work-up of a different unrelated disease. Out of the 243 cases, two developed MM within 3 years after sampling and 46 individuals were seen only at the time of sampling. IgM MGUS cases were excluded from the Heidelberg/Ulm cohort. For replication, 236/82 MGUS patients were identified for case-control/case-only replication in Essen within the Heinz -Nixdorf Recall (HNR) study (Schmermund et al. 2002). About 61% of the replication set were males with the mean age at diagnosis of 64 years, SD ± 9 years. Detection of MGUS was based on internationally accepted criteria (Criteria for the classification of monoclonal gammopathies 2003): monoclonal protein concentration less than 30 g/l, less than 10% monoclonal plasma cells in bone marrow, normal plasma calcium and kidney function and no bone destruction or anemia. The reference population for the Heidelberg/Ulm set consisted of 1285 German individuals from the HNR study of whom 59% were males with a mean age at sampling of 60 years, SD ± 8 years (Schmermund et al. 2002). The reference population for the Essen set was also recruited within the HNR study, adding up to 2484 individuals, not overlapping with the reference population for the Heidelberg set.

Illumina HumanOmniExpress-12v1.1 chip arrays were used for genotyping the Heidelberg/Ulm MGUS set and the corresponding control set was genotyped using the Illumina HumanOmniExpress-12v.1.0 chip array (Schmermund et al. 2002). The Essen set was genotyped using six different chips: 365 (15 cases, 350 controls) were genotyped on Illumina HumanCoreExome-12v1–1 chip arrays, 1491 (82 cases, 1409 controls) on Illumina HumanCoreExome-12 v1–0 chip arrays, 133 (119 cases, 14 controls) on Illumina Human660W Quad_v1 chip arrays, 811 (45 cases, 766 controls) on Illumina Human Omni-Quad V.1 chip arrays and 1385 (82 cases, 1303 controls) on Illumina HumanOmniExpress-12v.1.0 chip arrays (Additional file 1). The amount of overlaps among the SNPs genotyped is reported in Additional file 2. Quality control assessment for genotyping has previously been described and all variants and samples considered for the final analysis passed the predefined thresholds (Broderick et al. 2011; Chubb et al. 2013).

### Quality control of GWAS samples

We excluded SNPs with less than 1% minor allele frequency, and also if the call rate was less than 99% in cases and controls. Genotype distribution in controls was tested for Hardy-Weinberg equilibrium with a χ2 test with 1 degree of freedom or Fisher’s exact test and SNPs with *P* value lower than 10^− 5^ was removed. This stringent quality control filtering produced 489,555 autosomal SNPs from Heidelberg/Ulm GWAS set for further analysis common to all of the 243 cases and 1285 controls and also 82 HNR samples (cases) genotyped on the same array. Application of similar quality control protocol rendered 195,490 autosomal SNPs present in 236 cases and 2484 controls of Essen GWAS set.

### Statistical and bioinformatics analysis

#### Genome-wide case-control interaction analysis with CASSI and INTERSNP

Statistical analyses were performed with R version 3.3.1, CASSI version 3, and INTERSNP v1.15 (Table 1). All pairwise combinations of post-quality controlled selected SNPs from Heidelberg/Ulm GWAS were tested for interactions on genome-wide scale. The interaction term of a pairwise fixed effects logistic regression model involving binary regressors may be interpreted to be the ratio of the odds ratios of association between alleles in cases to the odds ratios of association between alleles in controls. Hence the odds ratio of association between alleles in cases will be equal to that of the population interaction odds ratio given the odds ratio of association between alleles in controls are equal to 1. Epistasis enforces a logistic regression with linearity assumption among the fixed single marker effects and interaction effect of the two markers (Karkkainen et al. 2015; Purcell et al. 2007). This includes all 489,555 SNPs and approximately N = 1.2 × 10^11^ interactions. However, linkage disequilibrium (LD) among the SNPs, if not taken into account in argument space, possibly renders a large portion of tests redundant which destabilizes the test statistic. To avoid this deflation of test power, we employed Wellek-Ziegler statistic which promises high variance stability and unbiased estimate on the condition of Hardy-Weinberg equilibrium conformity (Wellek and Ziegler 2009). CASSI Genome-Wide Interaction Analysis software is used to this end and we benefit from the computational efficiency thus achieved. Interaction test in CASSI (−wz) was applied with default initial pruning on single marker association *P* value at 10^− 3^ level of significance which decreases the computational burden (Ueki and Cordell 2012). This step selected 2.8 × 10^7^SNP pairs to be tested for interaction. For replication of interactions in the discovery set, reanalysis with full log-linear model was obtained with INTERSNP which selects a user-predefined number of top single marker hits as candidate SNPs for subsequent interaction tests. On a predefined level of 5000 selections, INTERSNP produced approximately N = 1.25 × 10^7^ variant pairs (Herold et al. 2009).

**Table 1:** Overview of tools and different subsequent protocols in use. Study designs enlist three stages of analysis

Tool in use	Statistic used	Statistical model in use	Default pre-selection criteria for interaction test	Study design	No. of tests performed	Bonferroni adjusted genome-wide level of significance (< 1% FDR)	No. of risk loci pairs discovered
CASSI	Wellek-Zeigler statistic	Logistic regression; fixed effects weighted model	Single marker test *P* value < 10^−3^	Discovery study	2.8 × 10^7^	5 × 10^− 10^	561
				Follow up study	4.4 × 10^5^	5 × 10^−10^	352
				Replication study	8.2 × 10^6^	5 × 10^−10^	23
INTERSNP	Chi square statistic	Full log-linear model	Top 5000 variants of single marker test	Discovery study	1.25 × 10^7^	8 × 10^−10^	none

*FDR* false discovery rate

#### Case-only analysis with CASSI

We chose the additional smaller cohort of 82 individuals with MGUS for a validation study and adopt a case-only approach. Case-only approach in SNP – SNP interaction studies answers the question of association (or correlation) between alleles of two loci irrespective of phenotypic categorization. Assuming the general population (control group) is devoid of any correlation between the specific loci, the case-only approach ensures evidence of gene-gene functional pair interactions with higher power in detection due to reduced number of multiple testing. As the risk loci are tested against each other rather than a reference set, allelic co-occurrence indicates bi-allelic interaction jointly predisposing phenotype changes (Ueki and Cordell 2012). However, case-only approach makes a quite strong assumption of no apparent genetic correlation among controls which is difficult to justify under the effects of LD which calls for cautious investigation of the results which further needs to be tested against a control population as reference to avoid misinterpretation due to the assumption. Case-only interaction test with CASSI with Wellek-Ziegler statistics (−wz-cc-only) on post quality-controlled SNPs rendered transformed Fisher’s Z statistics on 4.4 × 10^5^ out of a total of 1.2 × 10^11^ interaction pairs. We re-observed 31 genome-wide significant pairs from the discovery set. The number of overlaps detected between the two tested interaction population sets with high power extracted from the case-only set ensured viability of the replication GWAS data being used for replication (Wu et al. 2010).

#### Case-control replication study with CASSI

The Essen GWAS data consisting of 236 cases and 2484 controls was chosen for the case-control replication of the results obtained. The interaction tests for replication were performed similarly as explained before using Wellek-Ziegler statistic. Approximately N = 8.2 × 10^6^ tests were performed among the 195,490 SNPs after application of initial quality control. Among the quality-controlled SNPs from the Essen GWAS, we observed 188,198 overlaps with that of the Heidelberg/Ulm discovery GWAS. With approximately 35% cross table commonality we do not expect to see chance findings overlapping with high significance which takes care of the ‘winner’s curse’ (Shi et al. 2016; Poirier et al. 2015; Jiang and Yu 2016).

#### Network analysis with STRING

Two-locus epistasis evidence is verified by network construction with the in silico tool STRING. It is a web-based data repository dedicated to protein-protein interactions among 2.5 million proteins among 630 organisms including mammalians (version 8.0) (Jensen et al. 2009). Scores for each of the interacting protein pair is computed with combined scores integrating discovery probabilities from different sources adjusting for arbitrary false positives. Subsequently pathway enrichment is performed with the background repository for the interaction identified loci.

#### Gene prioritization and pathway analysis of the MGUS GWAS

Due to LD, causal regulatory elements often remain unidentified in genome-wide association studies. To gain biological insight, pathway analysis via gene-set enrichment is traditionally carried out to integrate signals from different sentinel variants and linked SNPs. We applied three tools designed for biological interpretation of GWAS: Pathway Scoring Algorithm (PASCAL), Data-driven Expression Prioritized Integration for Complex Traits (DEPICT) and Meta-Analysis Gene Set Enrichment of Variant Associations (MAGENTA). PASCAL is a pathway analysis tool developed for association summary statistics for variants annotated to genes. PASCAL uses maximum of chi-squares (MOCS) or sum of chi-squares (SOCS) statistics with null distribution as Gamma with varying degrees of freedom (Lamparter et al. 2016). Although pathway scoring was performed using both MOCS and SOCS statistics, the results were comparable and SOCS produced deflated significance levels with similar order as of that by MOCS. With empirical sampling and subsequent supervised clustering according to the significance levels introduced by single marker association tests, it utilizes the idea of gene fusions i.e. clusters of correlated genes, for which the variants are in LD. We performed gene set enrichment analysis (GSEA) using single marker *P* values from our published GWAS on MGUS and SNPs were mapped to closest genes searched 20 kb upstream and downstream from the gene. However, SNPs, corresponding to several genes responsible for regulating a single pathway, if they were in LD, were used to cluster the corresponding genes as single genetic entities for the given pathway and were called ‘*gene fusions*’. Kyoto encyclopedia of genes and genomes (KEGG), REACTOME and BioCarta libraries were used for pathway enrichment.

For further investigation of the distinguished pathways, we used DEPICT, a gene prioritization, tissue enrichment and pathway analysis tool for biological interpretation of GWASs distributed by Broad institute, which employs Python shell script on a Java platform for efficiency (Pers et al. 2015). This framework is built on sophisticated predictive modeling employing guilt by association on reconstituted gene sets to perform gene prioritization and GSEA (van Rheenen et al. 2016). Depict derives enrichment analysis viability from 77,840 gene expression datasets. We used DEPICT’s gene set knowledge base derived from Gene ontology (GO), Ensembl, The Mammalian Phenotype (MP), KEGG and REACTOME and followed analogous analyses.

Literature on pathway analysis with MAGENTA is populous (Koster et al. 2014; Wang et al. 2012; Duncan et al. 2014). We executed GSEA in MAGENTA from MATLAB platform as a replication tool. For pathway analysis, single marker association *P* values and chromosomal regions were annotated to genes corresponding to a pre-existing chromosomal range and enrichment computation was applied on non-confounders (Segrè et al. 2010; Shim et al. 2015). Pathway annotations were extracted in back end from GO, KEGG, Protein Analysis through Evolutionary Relationships (PANTHER), BioCarta and REACTOME databases. Across the three platforms thus used to prioritize pathways, *p* values are pooled subject to a total overlap and are combined using empirical Brown’s method. Traditionally p values thus analyzed are assumed to be dependent and thus the method applied provided a conservative restriction. All additional analyses were performed with R version 3.3.1.

## Results

### Genome-wide interaction analysis identifies 23 variant pairs

Two machine level in silico tools, CASSI and INTERSNP were employed to explore curated genome-wide interaction on a set of 243 German individuals diagnosed with MGUS in a case-control design. Among the 489,555 genotyped post-quality control SNPs, a brute search algorithm required 1.2 × 10^11^ tests at a whole-genome scale to perform a multivariate log-linear association test. However, CASSI restricted the runs to approximately 2.8 × 10^7^overall tests at the system-defined single marker association test threshold of P = 10^−3^. As approximately 2.8 × 10^7^ tests were performed, Bonferroni corrected level of global threshold of significance was determined to be 5 × 10^−10^ which restricts the family-wise error rate (FWER) at 1% (Table 1). Selection of chance findings over significant variants was thus avoided by applying Bonferroni correction for multiple testing. At this empirically determined *P* < 5 × 10^−10^, CASSI reported 561 significant variant pairs. The top ranked interaction (rs12471071 [2q37] - rs1385453 [9p24]) from the discovery set had a Wellek-Zeigler (W-Z) *P* = 4.19 × 10^−13^ and a simple logistic regression *P* = 8.67 × 10^−11^ (Additional file 3). Although the CASSI algorithm detected several common variant SNP pairs to be genome-wide significant, previous researches demonstrate that such findings were often subject to false discovery.

Interaction tests in INTERSNP were employed according to subjective pre-selection of the 5000 most significantly associated SNPs from the single marker tests (*P* = 2.47 × 10^−3^) which restricted the number of tests performed to an approximate 1.25 × 10^7^ pairs. Subsequently, 693 unique interaction pairs were identified at 5 × 10^−5^significance level where none of the observations reached genome-wide threshold of approximate 8 × 10^−10^ calculated taking Bonferroni correction into account on 99% confidence level. The top interaction was found to be between rs10099120 and rs3738270 with *P* = 9.05 × 10^−8^ and we note, rs10099120 is located in the intronic region of RALYL and rs3738270 corresponds to a missense mutation on IGFN1 (Additional file 4). Overall 52 common variant pairs were co-discovered for both INTERSNP and CASSI.

A follow-up case-only analysis by CASSI on the 82 cases genotyped, rendered approximately 4.4 × 10^5^ overall tests after initial single marker test shrinkage similar to that described above. The most significant interaction (rs4433825 [16p13] - rs2295179 [20p12]) showed a case-only P = 3.35 × 10^−24^ against W-Z case-control *P* = 1.5 × 10^−12^. At 5 × 10^−10^ level we detected 352 variant pairs replicated in the discovery set with varying levels of significance (Additional file 5). The order of significance observed in the follow-up analysis is consistent with the literature of case-only studies bolstering higher detection power due to the inherent mathematical assumption although we decided not to interpret the results as evidence of functional relation between variants due to the difference in number of overlapping SNP pairs tested with the discovery set. As the single marker pre-selection criteria prunes significant number of SNPs, the observed overlaps are ensured to have presumably higher individual fixed effects which is devoid of the hypotheses (Ueki and Cordell 2012). Nonetheless it confirms viability of the case-control replication study with a larger genetically overlapping sample(s).

**Fig. 1:**
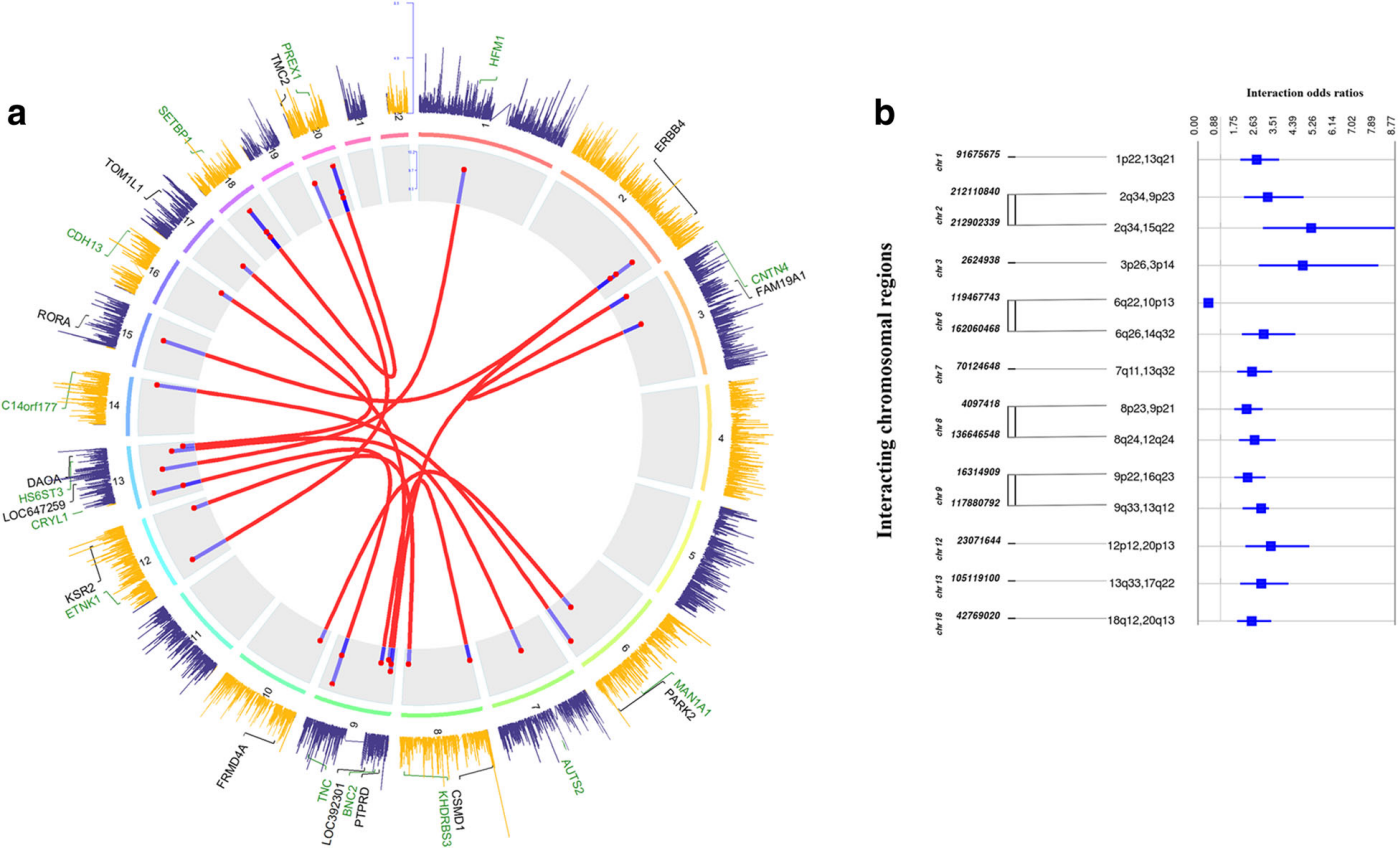
Interaction Analysis identifies 14 unique risk loci pairs. **a** Circos plot of genome-wide association and significant interaction results for the identified paired risk loci. The second outer most panel displays results from genome-wide association study on a Manhattan plot for autosomal variants on a log transformed scale (−*log*_10_) of 1 to 8.5. Negative log transformed interaction *P* values corresponding to each of the interaction pair is calculated from log linear transformed regression on the discovery set and is represented on an adjusted inflated scale of 9.3 to 10.2 in the second inner most panel. More than one unique variant pair combinations are present in the same interacting regions which are marked with their corresponding odds in this panel. Genome-wide significant paired loci are line-joined in the inner most panel based on their chromosomal positions (NCBI build 19 human genome). Annotations of single nucleotide polymorphisms to gene ids are displayed at the outer most panel. **b** Forest plot with embedded confidence intervals for each of the identified interaction pairs. Each pair indicates two interacting chromosomal locations with base pair information for the indexing loci. Paired variants annotated to the same indexing chromosomes are line joined. *chr* chromosome, *BP* base pair; OR, odds ratio; CI, confidence interval

Next, we evaluated W-Z interactions by CASSI in the case-control replication set consisting 8.2 × 10^6^ test pairs with an inflation factor of 1.0151 (Additional file 6). We were able to replicate 23 out of all 561 genome-wide significant variant pairs of the discovery set which are annotated to same chromosomal regions (Table 2, Fig. 1). The top interaction was found among variants annotated to TNC and CRYL1 corresponding to 9q33 and 13q12 (rs10118040 – rs7337130, W-Z P = 6.9 × 10^−11^ and rs1330368 – rs7337231, W-Z P = 2.48 × 10^−8^, respectively). Among the 23 replications, 14 were unique regions and there were 5 regions with multiple unique interactions. Interestingly, SETBP1 and PREX1 interaction at 18q12 and 20q13 were represented by 6 SNP-SNP pairwise overlaps with LD coefficient of r^2^ < 0.2 between SNPs belonging to corresponding regions. The locus at 20q13 has already been identified as a predisposing locus for MM and as an expression and methylation quantitative trait locus at PREX1 without affecting an active promoter site (Mitchell et al. 2016). SETBP1 is a well-established candidate gene harboring somatic mutation in various myeloid malignancies including secondary acute myeloid leukemia (sAML) and chronic myelomonocytic leukemia (CMML) (Makishima et al. 2013). Previously our group had identified 10 common variant risk loci for MGUS (Thomsen et al. 2017), among which two SNPs showed noteworthy interactions in our analysis: rs10251201 (7p21, GLCCI1) with rs1104869 (2p23, ALK), W-Z *P* = 8.75 × 10^−7^ and rs16966921 (18q12, GALNT1) with rs8092870 (18q12, CDH2), W-Z P = 1.71 × 10^−7^. Although both the latter two SNPs are located at 18q12, they are not in LD ( r^2^ < 0.2).

**Table 2:** Summary results for identified risk loci pair overlaps

				Discovery set								Replication set							
Gene1	Chr1	Gene2	Chr2	SNP1 (Risk allele)	Position (hg19,bp)	MAF	SNP2 (Risk allele)	Position (hg19,bp)	MAF	WZ *P*-value	OR (95% CI)	SNP1 (Risk allele)	Position (hg19,bp)	MAF	SNP2 (Risk allele)	Position (hg19,bp)	MAF	WZ *P* value	OR (95% CI)
TNC	9q33.1	CRYL1	13q12.11	rs10118040 (T)	117,879,414	0.40	rs7337130 (C)	21,021,343	0.31	**6.91E-11**	2.64 (1.91–3.65)	rs1330368 (A)	117,821,026	0.48	rs7337231 (G)	20,896,618	0.49	2.48E-08	1.05 (0.96–1.14)
SETBP1	18q12.3	PREX1	20q13.13	rs12959213 (C)	42,769,020	0.41	rs6066791 (T)	47,251,687	0.26	**7.07E-11**	2.39 (1.75–3.25)	rs11082429 (G)	42,743,790	0.44	rs170536 (A)	46,878,722	0.32	4.25E-08	1.01 (0.93–1.09)
SETBP1	18q12.3	PREX1	20q13.13	rs12959213 (C)	42,769,020	0.41	rs6066791 (T)	47,251,687	0.26	**7.07E-11**	2.39 (1.75–3.25)	rs1376230 (T)	42,703,052	0.35	rs6063251 (C)	47,015,157	0.43	6.37E-07	1.03 (0.94–1.11)
ERBB4	2q34	RORA	15q22.2	rs1546717 (G)	212,902,339	0.10	rs1159814 (A)	61,431,996	0.41	**9.07E-11**	5.03 (2.89–8.77)	rs6745249 (G)	213,130,571	0.48	rs974065 (A)	60,952,440	0.34	**1.06E-10**	1.13 (1.04–1.22)
PARK2	6q26	C14orf177	14q32.2	rs6455744 (T)	162,060,468	0.38	rs7359146 (C)	99,084,602	0.14	**1.12E-10**	2.92 (1.96–4.33)	rs6927285 (G)	162,010,329	0.43	rs8022922 (A)	98,987,292	0.44	**1.23E-14**	1.06 (0.98–1.14)
ETNK1	12p12.1	TMC2	20p13	rs2467112 (C)	23,071,644	0.19	rs1028441 (T)	2,600,186	0.24	**1.20E-10**	3.24 (2.12–4.95)	rs7313039 (C)	23,091,130	0.47	rs6050256 (T)	2,554,907	0.48	2.04E-07	1.05 (0.97–1.14)
^a^LOC646784 / HFM1	1p22.2	LOC647259	13q21.1	rs674135 (G)	91,675,675	0.26	rs4146191 (A)	62,872,965	0.47	**1.44E-10**	2.61 (1.89–3.60)	rs7416823 (T)	157,386,394	0.31	rs428328 (C)	63,110,606	0.41	2.24E-09	1.05 (0.96–1.13)
ERBB4	2q34	PTPRD	9p23	rs1437919 (A)	212,110,840	0.23	rs10978043 (G)	9,860,402	0.19	**2.64E-10**	3.38 (2.19–5.22)	rs6747637 (G)	212,406,789	0.45	rs4427223 (A)	10,663,815	0.48	**7.35E-14**	1.01 (0.93–1.09)
AUTS2	7p11.22	HS6ST3	13q32.1	rs1011780 (A)	70,124,648	0.28	rs9556582 (G)	97,040,531	0.46	**2.68E-10**	2.40 (1.75–3.29)	rs10267303 (T)	70,082,913	0.47	rs12876541 (C)	97,304,003	0.44	3.33E-08	1.06 (0.97–1.14)
SETBP1	18q12.3	PREX1	20q13.13	rs12959213 (C)	42,769,020	0.41	rs4810836 (T)	47,228,931	0.25	**3.04E-10**	2.40 (1.75–3.25)	rs11082429 (G)	42,743,790	0.44	rs170536 (A)	46,878,722	0.32	4.25E-08	0.98 (0.90–1.06)
SETBP1	18q12.3	PREX1	20q13.13	rs12959213 (C)	42,769,020	0.41	rs4810836 (T)	47,228,931	0.25	**3.04E-10**	2.40 (1.75–3.25)	rs1376230 (T)	42,703,052	0.35	rs6063251 (C)	47,015,157	0.43	6.37E-07	0.97 (0.89–1.05)
CNTN4	3p26.3	FAM19A1	3p14.1	rs2619566 (C)	2,624,938	0.12	rs1032376 (A)	68,317,975	0.19	**3.28E-10**	4.66 (2.71–8.02)	rs1499133 (C)	2,952,214	0.41	rs7610023 (T)	68,123,731	0.40	4.14E-09	1.05 (0.97–1.14)
CNTN4	3p26.3	FAM19A1	3p14.1	rs2619566 (G)	2,624,938	0.12	rs1032376 (A)	68,317,975	0.19	**3.28E-10**	4.66 (2.71–8.02)	rs1178491 (G)	2,342,825	0.36	rs6549098 (A)	68,323,280	0.40	2.83E-08	0.98 (0.90–1.06)
TNC	9q33.1	CRYL1	9q33.1	rs2071520 (T)	117,880,792	0.32	rs7337130 (C)	21,021,343	0.31	**3.50E-10**	2.80 (1.99–3.15)	rs1330368 (A)	117,821,026	0.48	rs7337231 (G)	20,896,618	0.49	2.48E-08	0.96 (0.89–1.04)
CSMD1	8p23.2	LOC392301	9q13	rs1700112 (G)	4,097,418	0.41	rs410684 (A)	31,673,588	0.42	**3.84E-10**	2.16 (1.62–2.87)	rs2740939 (C)	3,872,513	0.48	rs7853053 (T)	32,211,402	0.49	**2.04E-16**	1.04 (0.95–1.12)
CSMD1	8p23.2	LOC392301	9q13	rs1700112 (G)	4,097,418	0.41	rs410684 (A)	31,673,588	0.42	**3.84E-10**	2.16 (1.62–2.87)	rs2740929 (C)	3,879,918	0.49	rs7853053 (T)	32,211,402	0.49	**3.81E-10**	1.04 (0.95–1.12)
ERBB4	2q34	LOC729802	9p23	rs1437919 (A)	212,110,840	0.23	rs7851513 (G)	9,842,176	0.19	**3.92E-10**	3.10 (2.05–4.69)	rs6747637 (G)	212,406,789	0.45	rs4427223 (A)	10,663,815	0.48	**7.35E-14**	0.92 (0.85–0.99)
KHDRBS3	8q24.23	KSR2	12q24.23	rs4909494 (C)	136,646,548	0.46	rs10774941 (T)	118,037,655	0.27	**4.22E-10**	2.51 (1.83–3.45)	rs16905387 (G)	136,539,132	0.42	rs7972142 (A)	118,211,046	0.44	**3.97E-13**	1.05 (0.96–1.13)
SETBP1	18q12.3	PREX1	20q13.13	rs12959213 (C)	42,769,020	0.41	rs6095212 (T)	47,233,383	0.25	**4.25E-10**	2.39 (1.75–3.25)	rs11082429 (G)	42,743,790	0.44	rs170536 (A)	46,878,722	0.32	4.25E-08	1.04 (0.96–1.12)
SETBP1	18q12.3	PREX1	20q13.13	rs12959213 (C)	42,769,020	0.41	rs6095212 (T)	47,233,383	0.25	**4.25E-10**	2.39 (1.75–3.25)	rs1376230 (T)	42,703,052	0.35	rs6063251 (C)	47,015,157	0.43	6.37E-07	1.03 (0.95–1.11)
MAN1A1	6q22.31	FRMD4A	10p13	rs808034 (A)	119,467,743	0.39	rs789761 (C)	14,137,678	0.48	**4.72E-10**	0.46 (0.34–0.61)	rs1295392 (G)	119,676,177	0.45	rs751498 (A)	13,929,130	0.47	1.25E-09	1.05 (0.96–1.14)
BNC2	9p22.3	CDH13	16q23.3	rs7867771 (T)	16,314,909	0.28	rs11149564 (C)	83,441,027	0.44	**4.82E-10**	2.20 (1.62–3.00)	rs1415471 (A)	16,656,653	0.44	rs7194615 (G)	82,769,498	0.44	1.07E-08	1.02 (0.94–1.09)
DAOA	13q33.2	TOM1L1	17q22	rs5012127 (G)	105,119,100	0.17	rs4793773 (A)	52,646,414	0.27	**4.89E-10**	2.81 (1.88–4.02)	rs3015345 (A)	105,860,621	0.45	rs8070668 (G)	52,991,636	0.38	6.06E-10	1.05 (0.97–1.14)

Risk allele is the allele corresponding to which the test is performed and the odds ratio is calculated. Frequency of risk allele pair is tested against controls. SNP1 and SNP2 are the two SNP candidates of a pair from each population; gene1 and gene2are the corresponding genes annotated to SNP1 and SNP2, respectively. WZ p-value is Wellek Ziegler test *p*-value. OR, interaction odds ratio; MAF, minor allele frequency; bp, base pair; hg19, human genome build 19; CI, confidence interval

Bolding indicate genome wide significant observation at 99% level of significance. Underline indicate significant odds ratio

^a^denotes annotation of SNP to the two closest flanking genes

### ErbB signaling and B cell receptor signaling are enriched based on genetic network analysis

**Fig. 2:**
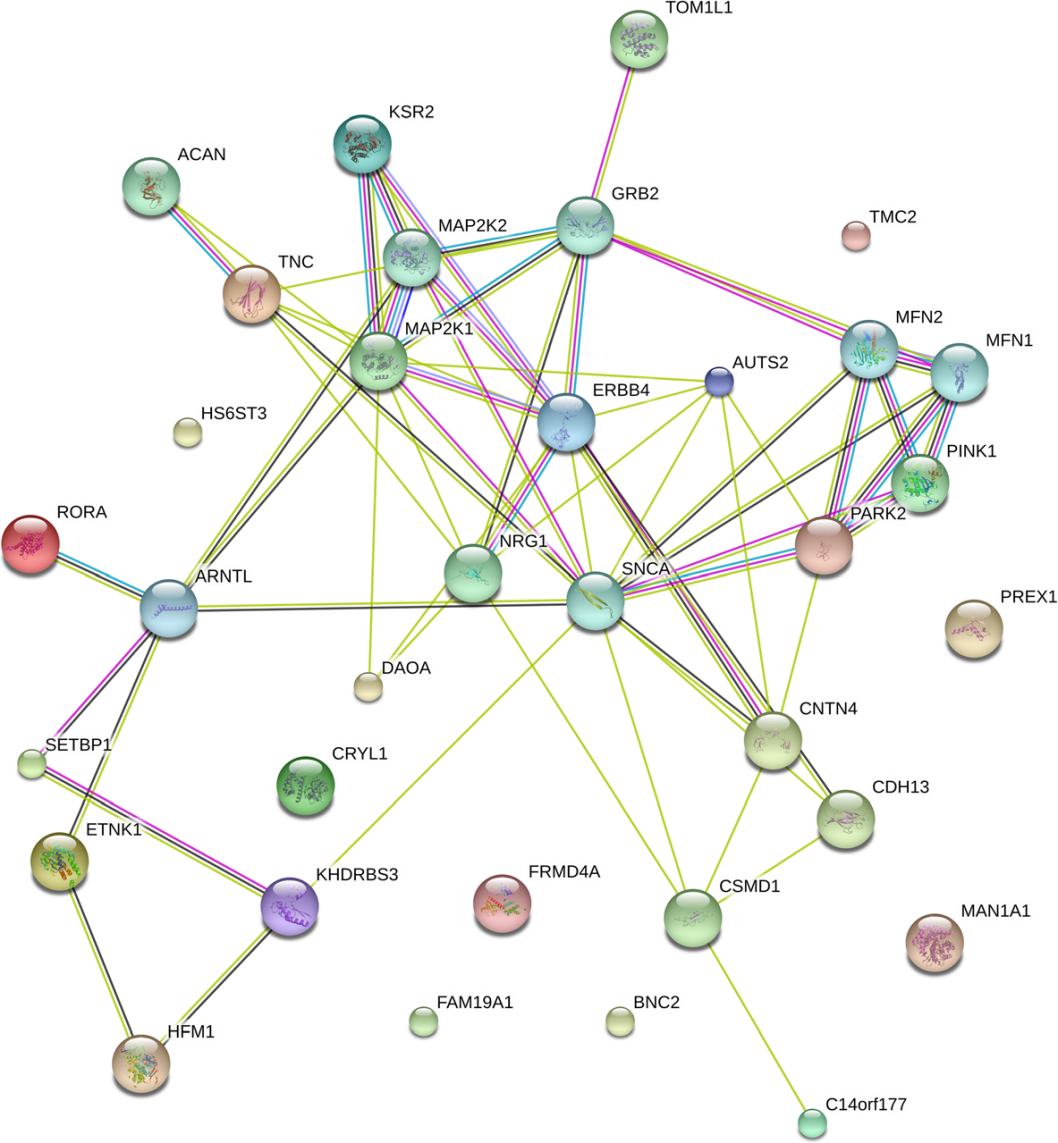
Genetic Interaction network. A network of 26 identified genes annotated to risk loci with added predicted genes in interaction. All nodes represent first order interaction. Colored edges convey status of predicted network edge correspondingly cyan, curated database; magenta, experimentally determined; forest green, gene neighborhood; red, gene fusion; navy blue, gene co-occurrence; lawn green, text mining; black, co-expression; lavender indigo, protein homology. Node color signifies protein functionality. Additional nodes are considered based on prediction score ≥ 0.9 (for more details, refer to STRING data base)

A partnership dependence structure of functional network was constructed with the risk variants from the final overlapping set subject to identifiable annotation from the interaction analyses. Twenty-six such reconstituted genes were used as nodes which together with first order interacting genes created scaffolding for further enrichment analysis (Fig. 2). Thirty-six potentially differentially regulated pathways were identified (Additional file 7). Among them were 18 enriched pathways at 0.01 level of significance, with as many as 5 gene nodes downstream to KEGG ErbB signaling pathway (*P* = 7.09 × 10^−5^) and 3 gene nodes downstream to KEGG B cell receptor signaling pathway (*P* = 5.32 × 10^−3^) were found to be the two most significant pathways.

### GSEA and pathway analysis confirms enrichment of ErbB cascade and detects pathways upstream to B cell receptor signaling in the MGUS GWAS

Similar to genome wide-association and interaction studies, pathway enrichment for MGUS is still unexplored in literature due to obvious limitations regarding caveats in identification and inclusion of adequate MGUS cases. We identified 65 enriched pathways at 95% confidence (19 at 99%) employing PASCAL (Additional file 8). For confirmation of our results we employed MAGENTA on the same set and detected 111 functionally enriched pathways significant at 95% level (22 at 99%) (Additional file 9). Although 28 overlapping pathways between the two algorithms used were discovered with a combined corrected *P* < 0.025 (Additional file 10), we wanted to extend our search introducing more detection power with curated microarray data. Further gene set enrichment analysis (GSEA) and pathway enrichment with DEPICT identified 99 pathways at the suggestive threshold of 10^−5^ (4 at genome-wide threshold of 5 × 10^−8^) (Additional file 11). With a combined analysis throughout the three algorithms, we observe 9 pathways with varying levels of significance (Table 3). Among the overlapping pathways, KEGG allograft rejection pathway (combined *P* = 1.60 × 10^−6^) and KEGG autoimmune thyroid disease pathway (combined P = 5.41 × 10^−6^), both related to B cell receptor signaling pathway, were the two most significant ones. Thus we determine that the B-cell receptor signaling pathway and EGFR regulatory pathway are present in both the interaction and genome-wide association study-oriented pathway analyses with statistical significance.

**Table 3:** Combined results of gene set enrichment analysis from MAGENTA, PASCAL and DEPICT. Pathways are pooled from several repositories which are enlisted with data base. *P* values from PASCAL, DEPICT and MAGENTA are corrected for multiple testing

		PASCAL	DEPICT	MAGENTA	
Data base	Pathway	*P* value	*P* value	*P* value	*Combined *P* value
KEGG	Allograft rejection	0.083	0.001	0.005	5.62E-04
KEGG	Autoimmune thyroid disease	0.043	0.001	0.022	9.30E-04
KEGG	Glycosaminoglycan biosynthesis keratan sulfate	0.008	0.171	0.036	9.89E-03
REACTOME	Platelet aggregation plug formation	0.044	0.952	0.005	2.28E-02
REACTOME	EGFR downregulation	0.776	0.951	0.003	2.45E-02
REACTOME	Integrin cell surface interactions	0.16	0.396	0.011	4.51E-02
KEGG	Dorso ventral axis formation	0.151	0.233	0.022	4.78E-02
REACTOME	P130CAS linkage to MAPK signaling for integrins	0.035	0.988	0.032	4.85E-02

*Pooled *p* values are combined using empirical Brown’s method assuming dependency across test hypotheses

## Discussion

Here we investigated MGUS, the preliminary phase of the lymphoproliferative neoplasm MM, assuming biological evidence of genetic burden to be spread across the whole genome spectrum. Performing genome-wide interaction analysis with case/control and case-only data together with subsequent follow-ups, our design essentially narrows down brute-force search to rather sizable genomic regions. Extending from the methodology developed by Ueki and colleagues for systematic implementation of Wellek – Zeigler statistics in interaction tests (Ueki and Cordell 2012), we propose a workflow to integrate statistical findings with biological knowledge base. Streamlining detection of risk loci with enriched protein-protein interacting networks to discover differentially regulated novel pathways facilitates understanding of disease mechanisms and congregates statistical evidence with biologically interpretable information.

Rather than simple bi-allelic association estimated with a dichotomous logistic model, we employed Pearson’s product-moment correlation coefficient r^2^ for association test. In simple linear association models, loss in precision is surmountable subject to non-conformity to Hardy-Weinberg equilibrium, especially while estimating for variant pairs in high LD. Wellek and Zeigler established genotype-based estimator of pearsonian r to be unbiased and circumstantial loss in information for unphased genotypes to be considerably lower than the haplotype-based estimators (Wellek and Ziegler 2009). Hence, we used genotype-based W-Z statistic, which employs a variance stabilizing Fisher’s z transformation and produces robust estimates with high convergence rate.

Literature recognizes statistical interaction and functional interaction among genes/proteins exclusively, as the former stands for genetic association and the latter signifies biological process dependency. Thus, translation of statistical evidence to interpretable genetic functional involvement is of utmost importance. Creating node-based protein networks with STRING enabled us to visualize statistical clusters of genes in inter-play among the interacting genomic loci and observe the enriched biological processes that confer to them relating small genetic hubs created by clusters of genes specific to pathways. Consequently, we executed gene-prioritization and enrichment analysis accumulating tests in the previously published MGUS GWAS data from three different algorithms, PASCAL, MAGENTA and DEPICT, that engage different repositories. Observed overlaps therefore served as strong evidence connecting interacting common variant loci to the pathways via genetic networks.

We detected KEGG allograft rejection (hsa05330) and autoimmune thyroid disease (hsa05320) pathways with highest combined power from all three GWAS enrichment algorithms. Conspicuously B cell receptor signaling which is enriched in our genetic network is also downstream to both of the pathways. This pathway is shown to be dependent on mitogen-activated protein kinase signaling (MAPK), phosphatidylinositol-3 kinase and protein kinase B signaling (PI3K-Akt), nuclear factor κB signaling (NF-κB) and calcium signaling pathways. It is also dependent on adhesion molecule induced mechanisms that mediate immune tolerance in T cell receptor signaling cascade. Detection of ErbB signaling (hsa04012) in genetic network enrichment is also supported by detection of EGFR downregulation pathway from our pathway analysis. We identified ErbB4 (HER4) to be a high-risk locus interacting with 15q22 and 9p23. ErbB4 is one of the four epidermal growth factor receptor (EGFR) family members with tyrosine kinase activity. In both cancerous and non-cancerous cells, EGFR plays a crucial role in controlling key cellular pathways influencing cell proliferation, differentiation and development through MAPK and PI3K/Akt pathways and overexpression of which is associated to multiple cancer types (Yarden and Sliwkowski 2001). *PTPRD* has been characterized as a tumor suppressor gene in MM and a homozygous deletion in PTPRD encoding locus is known to modulate phosphorylation of STAT3 that promotes IL6 signaling (Lohr et al. 2014). Whereas, RORɑ is a regulator of circadian clock, responsible for cytokine secretion especially interleukins (Paiva et al. n.d.). Contextually, with a mechanistic aggression, circulating MM tumor cells undergo circadian rhythm dependent selective egression. Circadian rhythm was one of the suggestively selected enriched pathways encompassing the genetic network (Additional file 7, *p* = 1.38 × 10^−2^). Involvement of HER4 in cancer has yet not been comprehensively addressed, although it has been shown to be overexpressed in colorectal cancer and postulated to promote carcinogenesis in general (Lau et al. 2014; Williams et al. n.d.).

We find rs10251201, one of the previously identified risk loci for MGUS with moderate significance, annotated to 36 kb 5′ to GLCCT1 in interaction with rs1104869 mapping to an intronic region of an oncogene, anaplastic lymphoma receptor tyrosine kinase (ALK). Notably ALK amplification, mutations and especially chromosomal rearrangements have been found in several cancers (Chiarle et al. 2008). Another previously identified MGUS risk locus (rs16966921) annotated to GALNT1 was shown in our data to have moderately significant interaction with (rs8092870) annotated to cadherin 2 (CDH2), an adhesion molecule and downstream target of FGFR3 signaling pathway (Takehara et al. 2015). Cell adhesion is an integral part of cell surface interaction and is of two major types: cell-to-cell and cell-to-extracellular matrix (ECM). Cadherin family cell adhesion molecules play important roles in the formation and functions of cell-cell adhesions. CDH2 is associated with several neoplasias and has been reported to be overexpressed in MM co-existing with the t(4,14) translocation (Dring et al. 2004). Interestingly, we also identified a novel risk locus on another cadherin gene, CDH13 in an interaction with 9p22. CDH13 is not involved in cell-cell adhesion, but protects vascular endothelial cells from apoptosis due to oxidative stress and is found to be hypermethylated in myeloid leukemia, B-cell lymphomas among several other cancers (Andreeva and Kutuzov 2010).

At cell-ECM adhesions, the major transmembrane proteins are integrin heterodimers. Several integrins, including integrin-β1, −β7 and -α8 have been shown to play a role in MM cell adhesion, migration, invasion, bone marrow homing and drug resistance. We observed REACTOME integrin cell surface interactions pathway as a comparably significant hit from all three of our pathway analyses. Our assumption of interplay between cell adhesion and integrin pathways is also supported by the discovery of the REACTOME platelet aggregation (plug information) pathway, a crucial adhesion mechanism not only in normal hemostasis but also in pathophysiological processes such as inflammation, immune-mediated host defense and cancer metastasis. Integrin-αIIbβ3 plays a key role in platelet adhesion and aggregation (Ruggeri and Mendolicchio 2007).

Functional epistasis traditionally derives its appeal on the assumption of polygenic risk where genes in ensemble are supposed to accumulate larger deregulating impact than considered individually. Although direct aggregation of interaction test and enrichment analysis may seem tempting following a step-wise implementation procedure, caveats due to low statistical power render it unfeasible. Hence, a major limitation of this study is the low case numbers which mostly is due to the scarcity of identifiable individuals. MGUS being an asymptomatic condition, studies are dependent on indirect diagnosis in its entirety. We admit that detection power of our analysis is marginally compromised accounting for stringent selection criteria since pairwise interaction algorithms require large statistical power to avoid high false discovery rate and that may result subsequent rejection of false negative results. However, we have minimized this loss of information by analyzing gene set and pathway enrichment on GWAS summary statistics parallel to the interaction test. Fang et al. 2017 have recently proposed such a procedure on building pathway map based on linear interaction model. Although to make the later analysis viable, their study proposes selection of risk loci at a very nominal level of significance (Fang et al. 2017). Unfortunately, this rather permissive selection criterion makes their proposed BridGE algorithm unreliable as it may allow a large proportion of false positive findings. This calls for further careful inspection in the statistics to achieve robustness in inference.

## Conclusion

In summary, we developed a method that unifies variant pair interaction with genetic networks and pathway enrichment. We also discovered evidence which supports that several signaling cascades including B cell receptor, epidermal growth receptor and cell adhesion related pathways play a role and are regulated via several interacting loci in development of MGUS that possibly explicates its further progression to MM.

## Additional files

Additional file 1: Top interactions from W-Z discovery set interaction test compared with simple logistic linear interaction test (brute force epistasis). Description: SNP1 and SNP2 are the two SNP candidates of a pair from the discovery population belonging to chromosomes denote by Chr1 and Chr2; gene1 and gene2 are the corresponding genes annotated to SNP1 and SNP2, respectively. W-Z *P* value is Wellek Ziegler test *p*-value. (DOCX 22 kb)

Additional file 2: Top interactions from W-Z case-only test in confirmation set. Description: SNP1 and SNP2 are the two corresponding SNP candidates of a pair from the case-only population belonging to chromosomes denote by Chr1 and Chr2; gene1 and gene2 are the corresponding genes annotated to SNP1 and SNP2, respectively. W-Z *P* value is Wellek Ziegler case-only test *p*-value; BP is base pair. (DOCX 21 kb)

Additional file 3: Top interactions from W-Z interaction test on the replication set. Description: SNP1 and SNP2 are the two SNP candidates of a pair from the replication set population belonging to chromosomes denote by Chr1 and Chr2; gene1 and gene2 are the corresponding genes annotated to SNP1 and SNP2, respectively. W-Z *P* value is Wellek Ziegler case-control test *p*-value; BP is base pair. (DOCX 22 kb)

Additional file 4: Overlapped interactions from INTERSNP and CASSI W-Z interaction tests on discovery set. Description: SNP1 and SNP2 are the two SNP candidates of a pair from the discovery set population belonging to chromosomes denote by Chr1 and Chr2; gene1 and gene2 are the corresponding genes annotated to SNP1(s) and SNP2(s), respectively. W-Z *P* value is Wellek Ziegler case-control test *p*-value from CASSI and INTERSNP P value is full log-linear test *p*-value from INTERSNP; BP is base pair. IN: INTERSNP; CAS: CASSI. (DOCX 22 kb)

Additional file 5: Gene set enrichment analysis in genetic network with STRING. Description: Based on the indexing nodes and the additional predicted first order interacting m=nodes, STRING performs enrichment analysis on several molecular, biological, cellular process related pathway analysis with Gene Onltology (GO) and KEGG database. All tests are performed with guilt by association assumption and *P* values are corrected for multiple testing. A protein-protein enrichment index is reported with analysis depicting level of confidence in the detected enriched processes which is reported to be 0.0039 (significant at 5% level). (DOCX 21 kb)

Additional file 6: PASCAL gene set enrichment analysis results at 1% level of significance. (DOCX 20 kb)

Additional file 7: MAGENTA gene set enrichment analysis results at 1% level of significance. (DOCX 20 kb)

Additional file 8: All detected pathways mutually discovered in both MAGENTA and PASCAL at a 5% level of combined significance. (DOCX 21 kb)

Additional file 9: DEPICT gene set enrichment analysis results at Bonferroni corrected genome wide significance level. (DOCX 18 kb)

Additional file 10: Summary of Illumina bead chips used for genotyping different batches of cases and controls. (DOCX 19 kb)

Additional file 11: Number of overlaps in number of SNPs prior quality control between different chips used in genotyping. Chip numbers are defined in Additional file 10. (DOCX 19 kb)

## Abbreviations

CMML: Chronic myelomonocytic leukemia
EGFR: Epidermal growth factor receptor
FWER: Family wise error rate
GO: Gene ontology
GSEA: Gene set enrichment analysis
HNR: Heinz Nixdorf-Recall
KEGG: Kyoto encyclopedia of genes and genomes
LD: Linkage disequilibrium
MGUS: Monoclonal gammopathy of undetermined significance
MM: Multiple myeloma
MOCS: Maximum of chi-square
MP: Mammalian Phenotype
PANTHER: Protein Analysis through Evolutionary Relationships
sAML: Secondary acute myeloid leukemia
SNP: Single nucleotide polymorphism
SOCS: Sum of chi-square
W-Z: Wellek-Zeigler

## References

[CR1] Andreeva AV, Kutuzov MA (2010). Cadherin 13 in cancer. Genes Chromosom Cancer.

[CR2] Broderick P, Chubb D, Johnson DC, Weinhold N, Forsti A, Lloyd A, Olver B, Ma YP, Dobbins SE, Walker BA (2011). Common variation at 3p22.1 and 7p15.3 influences multiple myeloma risk. Nat Genet.

[CR3] Chiarle R, Voena C, Ambrogio C, Piva R, Inghirami G (2008). The anaplastic lymphoma kinase in the pathogenesis of cancer. Nat Rev Cancer.

[CR4] Chubb D, Weinhold N, Broderick P, Chen B, Johnson DC, Forsti A, Vijayakrishnan J, Migliorini G, Dobbins SE, Holroyd A (2013). Common variation at 3q26.2, 6p21.33, 17p11.2 and 22q13.1 influences multiple myeloma risk. Nat Genet.

[CR5] Cordell HJ (2009). Detecting gene-gene interactions that underlie human diseases. Nat Rev Genet.

[CR6] Criteria for the classification of monoclonal gammopathies (2003). Multiple myeloma and related disorders: a report of the international myeloma working group. Br J Haematol.

[CR7] Dispenzieri A, Katzmann JA, Kyle RA, Larson DR, Melton LJ, Colby CL, Therneau TM, Clark R, Kumar SK, Bradwell A (2010). Prevalence and risk of progression of light-chain monoclonal gammopathy of undetermined significance: a retrospective population-based cohort study. Lancet (London, England).

[CR8] Dring AM, Davies FE, Fenton JAL, Roddam PL, Scott K, Gonzalez D, Rollinson S, Rawstron AC, Rees-Unwin KS, Li C (2004). A global expression-based analysis of the consequences of the t(4;14) translocation in myeloma. Clin Cancer Res.

[CR9] Duncan LE, Holmans PA, Lee PH, O'Dushlaine CT, Kirby AW, Smoller JW, Öngür D, Cohen BM (2014). Pathway analyses implicate glial cells in schizophrenia. PLoS One.

[CR10] Fang G, Wang W, Paunic V, Heydari H, Costanzo M, et al. Discovering genetic interactions bridging pathways in genome-wide association studies. bioRxiv 182741. (2017). 10.1101/182741. 10.1038/s41467-019-12131-7 PMC6753138 31537791

[CR11] Frank C, Fallah M, Chen T, Mai EK, Sundquist J, Forsti A, Hemminki K (2016). Search for familial clustering of multiple myeloma with any cancer. Leukemia.

[CR12] Greenberg AJ, Lee AM, Serie DJ, McDonnell SK, Cerhan JR, Liebow M, Larson DR, Colby CL, Norman AD, Kyle RA (2013). Single-nucleotide polymorphism rs1052501 associated with monoclonal gammopathy of undetermined significance and multiple myeloma. Leukemia.

[CR13] Greenberg AJ, Rajkumar SV, Vachon CM (2012). Familial monoclonal gammopathy of undetermined significance and multiple myeloma: epidemiology, risk factors, and biological characteristics. Blood.

[CR14] Herold C, Steffens M, Brockschmidt FF, Baur MP, Becker T (2009). INTERSNP: genome-wide interaction analysis guided by a priori information. Bioinformatics.

[CR15] Jensen LJ, Kuhn M, Stark M, Chaffron S, Creevey C, Muller J, Doerks T, Julien P, Roth A, Simonovic M (2009). STRING 8—a global view on proteins and their functional interactions in 630 organisms. Nucleic Acids Res.

[CR16] Jiang W, Yu W (2016). Power estimation and sample size determination for replication studies of genome-wide association studies. BMC Genomics.

[CR17] Karkkainen HP, Li Z, Sillanpaa MJ (2015). An efficient genome-wide multilocus epistasis search. Genetics.

[CR18] Khatri P, Sirota M, Butte AJ (2012). Ten years of pathway analysis: current approaches and outstanding challenges. PLoS Comput Biol.

[CR19] Koster R, Mitra N, D'Andrea K, Vardhanabhuti S, Chung CC, Wang Z, Loren Erickson R, Vaughn DJ, Litchfield K, Rahman N (2014). Pathway-based analysis of GWAs data identifies association of sex determination genes with susceptibility to testicular germ cell tumors. Hum Mol Genet.

[CR20] Kyle RA, Larson DR, Therneau TM, Dispenzieri A, Kumar S, Cerhan JR, Rajkumar SV (2018). Long-term follow-up of monoclonal Gammopathy of undetermined significance. N Engl J Med.

[CR21] Kyle RA, Therneau TM, Rajkumar SV, Larson DR, Plevak MF, Offord JR, Dispenzieri A, Katzmann JA, Melton LJ (2006). Prevalence of monoclonal gammopathy of undetermined significance. N Engl J Med.

[CR22] Lamparter D, Marbach D, Rueedi R, Kutalik Z, Bergmann S (2016). Fast and rigorous computation of gene and Pathway scores from SNP-based summary statistics. PLoS Comput Biol.

[CR23] Landgren O, Kristinsson SY, Goldin LR, Caporaso NE, Blimark C, Mellqvist UH, Wahlin A, Bjorkholm M, Turesson I (2009). Risk of plasma cell and lymphoproliferative disorders among 14621 first-degree relatives of 4458 patients with monoclonal gammopathy of undetermined significance in Sweden. Blood.

[CR24] Lau C, Killian KJ, Samuels Y, Rudloff U: ERBB4 Mutation Analysis: Emerging Molecular Target for Melanoma Treatment. In: Thurin M., Marincola F. (eds) Molecular Diagnostics for Melanoma. Methods in Molecular Biology (Methods and Protocols). Totowa: Humana Press. 2014; 1102. 10.1007/978-1-62703-727-3_24 PMC5125540 24258993

[CR25] Lee YH, Gyu Song G (2015). Genome-wide pathway analysis in pancreatic cancer. J Buon.

[CR26] Lohr JG, Stojanov P, Carter SL, Cruz-Gordillo P, Lawrence MS, Auclair D, Sougnez C, Knoechel B, Gould J, Saksena G (2014). Widespread genetic heterogeneity in multiple myeloma: implications for targeted therapy. Cancer Cell.

[CR27] Makishima H, Yoshida K, Nguyen N, Przychodzen B, Sanada M, Okuno Y, Ng KP, Gudmundsson KO, Vishwakarma BA, Jerez A (2013). Somatic SETBP1 mutations in myeloid malignancies. Nat Genet.

[CR28] Manolio TA, Collins FS, Cox NJ, Goldstein DB, Hindorff LA, Hunter DJ, McCarthy MI, Ramos EM, Cardon LR, Chakravarti A (2009). Finding the missing heritability of complex diseases. Nature.

[CR29] Mitchell JS, Li N, Weinhold N, Forsti A, Ali M, van Duin M, Thorleifsson G, Johnson DC, Chen B, Halvarsson BM (2016). Genome-wide association study identifies multiple susceptibility loci for multiple myeloma. Nat Commun.

[CR30] Paiva B, Paino T, Sayagues JM, Garayoa M, San-Segundo L, Martín M, Mota I, Sanchez ML, Bárcena P, Aires-Mejia I, Corchete L, Jimenez C, Garcia-Sanz R, Gutierrez NC, Ocio EM, Mateos MV, Vidriales MB, Orfao A, San Miguel JF, et al: Detailed characterization of multiple myeloma circulating tumor cells shows unique phenotypic, cytogenetic, functional, and circadian distribution profile. (1528–0020 (Electronic)). n.d. 10.1182/blood-2013-06-510453 24072855

[CR31] Pers TH, Karjalainen JM, Chan Y, Westra H-J, Wood AR, Yang J, Lui JC, Vedantam S, Gustafsson S, Esko T et al: Biological interpretation of genome-wide association studies using predicted gene functions. 2015, 6:5890. 10.1038/ncomms6890 PMC4420238 25597830

[CR32] Poirier JG, Faye LL, Dimitromanolakis A, Paterson AD, Sun L, Bull SB (2015). Resampling to address the Winner’s curse in genetic association analysis of time to event. Genet Epidemiol.

[CR33] Purcell S, Neale B, Todd-Brown K, Thomas L, Ferreira MA, Bender D, Maller J, Sklar P, de Bakker PI, Daly MJ (2007). PLINK: a tool set for whole-genome association and population-based linkage analyses. Am J Hum Genet.

[CR34] Ramanan VK, Shen L, Moore JH, Saykin AJ (2012). Pathway analysis of genomic data: concepts, methods, and prospects for future development. Trends Genet.

[CR35] Ruggeri ZM, Mendolicchio GL (2007). Adhesion mechanisms in platelet function. Circ Res.

[CR36] Schmermund A, Mohlenkamp S, Stang A, Gronemeyer D, Seibel R, Hirche H, Mann K, Siffert W, Lauterbach K, Siegrist J (2002). Assessment of clinically silent atherosclerotic disease and established and novel risk factors for predicting myocardial infarction and cardiac death in healthy middle-aged subjects: rationale and design of the Heinz Nixdorf RECALL study. Risk factors, evaluation of coronary calcium and lifestyle. Am Heart J.

[CR37] Segrè AV, Consortium D, Investigators M, Groop L, Mootha VK, Daly MJ, Altshuler D (2010). Common inherited variation in mitochondrial genes is not enriched for associations with type 2 diabetes or related glycemic traits. PLoS Genet.

[CR38] Shi J, Park JH, Duan J, Berndt ST, Moy W, Yu K, Song L, Wheeler W, Hua X, Silverman D (2016). Winner's curse correction and variable Thresholding improve performance of polygenic risk modeling based on genome-wide association study summary-level data. PLoS Genet.

[CR39] Shim U, Kim HN, Lee H, Oh JY, Sung YA, Kim HL (2015). Pathway analysis based on a genome-wide association study of polycystic ovary syndrome. PLoS One.

[CR40] Takehara T, Teramura T, Onodera Y, Frampton J, Fukuda K. Cdh2 stabilizes FGFR1 and contributes to primed-state pluripotency in mouse epiblast stem cells. Scientific Reports. 2015;5(1). 10.1038/srep14722 PMC4588589 26420260

[CR41] Thomsen H, Campo C, Weinhold N, Filho MI, Pour L, Gregora E, Vodicka P, Vodickova L, Hoffmann P, Nöthen MM, Jöckel KH, Langer C, Hajek R, Goldschmidt H, Hemminki K, Försti A. Genomewide association study on monoclonal gammopathy of unknown significance (MGUS). Eur J Haematol. 2017;99(1):70–9. 10.1111/ejh.12892 28375557

[CR42] Ueki M, Cordell HJ (2012). Improved statistics for genome-wide interaction analysis. PLoS Genet.

[CR43] van Rheenen W, Shatunov A, Dekker AM, McLaughlin RL, Diekstra FP, Pulit SL, van der Spek RAA, Vosa U, de Jong S, Robinson MR (2016). Genome-wide association analyses identify new risk variants and the genetic architecture of amyotrophic lateral sclerosis. Nat Genet.

[CR44] Wang J, Gao F, Liu Z, Qiao M, Niu X, Zhang K-Q, Huang X (2012). Pathway and molecular mechanisms for malachite green biodegradation in Exiguobacterium sp. MG2. PLoS One.

[CR45] Weinhold N, Johnson DC, Rawstron AC, Forsti A, Doughty C, Vijayakrishnan J, Broderick P, Dahir NB, Begum DB, Hosking FJ (2014). Inherited genetic susceptibility to monoclonal gammopathy of unknown significance. Blood.

[CR46] Wellek S, Ziegler A (2009). A genotype-based approach to assessing the association between single nucleotide polymorphisms. Hum Hered.

[CR47] Williams CS, Bernard JK, Demory Beckler M, Almohazey D, Washington MK, Smith JJ, Frey MR: ERBB4 is over-expressed in human colon cancer and enhances cellular transformation. (1460–2180 (Electronic)). n.d. 10.1093/carcin/bgv049 PMC4572918 25916654

[CR48] Wu X, Dong H, Luo L, Zhu Y, Peng G, Reveille JD, Xiong M (2010). A novel statistic for genome-wide interaction analysis. PLoS Genet.

[CR49] Yarden Y, Sliwkowski MX (2001). Untangling the ErbB signalling network. Nat Rev Mol Cell Biol.

[CR50] Yi S, Lin S, Li Y, Zhao W, Mills GB, Sahni N (2017). Functional variomics and network perturbation: connecting genotype to phenotype in cancer. Nat Rev Genet.

