# Supplementary material for: Enrichment of B cell receptor signaling and epidermal growth factor receptor pathways in monoclonal gammopathy of undetermined significance: a genome-wide genetic interaction study
Source: Mol Med. 2018 Jun 11;24:30. doi: 10.1186/s10020-018-0031-8 (PMC6016882; doi:10.1186/s10020-018-0031-8)
Supplement: Supplementary file 1 — Top interactions from W-Z discovery set interaction test compared with simple logistic linear interaction test (brute force epistasis). Description: SNP1 and SNP2 are the two SNP candidates of a pair from the discovery population belonging to chromosomes denote by Chr1 and Chr2; gene1 and gene2 are the corresponding genes annotated to SNP1 and SNP2, respectively. W-Z P value is Wellek Ziegler test p-value. (DOCX 22 kb) [file 10020_2018_31_MOESM1_ESM.docx]

**Additional file 1.** Top interactions from W-Z discovery set interaction test compared with simple logistic linear interaction test (brute force epistasis). SNP1 and SNP2 are the two SNP candidates of a pair from the discovery population belonging to chromosomes denote by Chr1 and Chr2; gene1 and gene2 are the corresponding genes annotated to SNP1 and SNP2, respectively. W-Z P value is Wellek Ziegler test p-value.

| **gene1** | **Chr1** | **SNP1** | **gene2** | **Chr2** | **SNP2** | **W-Z P value** | **Logistic regression**  **P value** |
| --- | --- | --- | --- | --- | --- | --- | --- |
| OR6B3 | 2 | rs12471071 | CDC37L1 | 9 | rs1385453 | 4.19E-13 | 8.67E-11 |
| LOC729108 | 3 | rs7652856 | LOC727862 | 17 | rs8074928 | 6.63E-13 | 8.97E-10 |
| C1orf129 | 1 | rs4656817 | hCG 1820717 | 13 | rs1326701 | 6.67E-13 | 1.62E-10 |
| ACVR2A | 2 | rs12691767 | TRBV20OR9-2 | 9 | rs855508 | 1.18E-12 | 1.82E-08 |
| LOC730216 | 7 | rs292661 | CSMD1 | 8 | rs4875730 | 1.21E-12 | 4.37E-10 |
| ABHD5 | 3 | rs4682696 | PPFIBP1 | 12 | rs7958124 | 1.25E-12 | 2.18E-10 |
| PRELID2 | 5 | rs6580355 | LOC390299 | 12 | rs33233 | 1.65E-12 | 3.57E-11 |
| SLC13A1 | 7 | rs4731094 | LOC390829 | 18 | rs2077149 | 1.65E-12 | 6.39E-10 |
| OR6B3 | 2 | rs12471071 | CDC37L1 | 9 | rs6476893 | 2.02E-12 | 2.72E-10 |
| BARX1 | 9 | rs4344139 | LOC347292 | 9 | rs1885968 | 2.22E-12 | 2.12E-08 |
| MYEOV2 | 2 | rs1992307 | CDC37L1 | 9 | rs1385453 | 2.62E-12 | 6.94E-10 |
| GRIA2 | 4 | rs17246641 | ZFAND3 | 6 | rs6933547 | 3.05E-12 | 6.99E-08 |
| MCAM | 11 | rs2249466 | CCL23 | 17 | rs854666 | 3.31E-12 | 3.97E-08 |
| CRISPLD1 | 8 | rs2954870 | CDKN3 | 14 | rs4898835 | 3.68E-12 | 2.17E-09 |
| PI15 | 8 | rs2731995 | CDKN3 | 14 | rs4898835 | 3.93E-12 | 1.84E-09 |
| HABP2 | 10 | rs4918844 | OTOR | 20 | rs4814551 | 7.00E-12 | 1.05E-07 |
| IHPK3 | 6 | rs568901 | TRPC6 | 11 | rs1230960 | 7.22E-12 | 7.61E-10 |
| EFR3B | 2 | rs7575363 | MACROD2 | 20 | rs716316 | 7.64E-12 | 3.24E-09 |
| GJB5 | 1 | rs4653061 | OLFML2B | 1 | rs2490431 | 8.08E-12 | 1.18E-08 |
| MYEOV2 | 2 | rs1992307 | CDC37L1 | 9 | rs6476893 | 9.37E-12 | 1.52E-09 |
| MAN2A1 | 5 | rs185088 | XRCC1 | 19 | rs3213356 | 9.95E-12 | 4.43E-09 |
| LOC645521 | 2 | rs13383210 | tcag7.893 | 7 | rs12672973 | 1.10E-11 | 7.37E-09 |
| MAN2A1 | 5 | rs185088 | ZNF575 | 19 | rs2030404 | 1.10E-11 | 4.82E-09 |
| ABHD5 | 3 | rs4682696 | PPFIBP1 | 12 | rs7966058 | 1.18E-11 | 8.04E-10 |
| BARX1 | 9 | rs3996253 | LOC347292 | 9 | rs1885968 | 1.32E-11 | 4.19E-09 |
| AGTPBP1 | 9 | rs11141010 | ST8SIA2 | 15 | rs11632278 | 1.34E-11 | 4.17E-09 |
