# Supplementary material for: Enrichment of B cell receptor signaling and epidermal growth factor receptor pathways in monoclonal gammopathy of undetermined significance: a genome-wide genetic interaction study
Source: Mol Med. 2018 Jun 11;24:30. doi: 10.1186/s10020-018-0031-8 (PMC6016882; doi:10.1186/s10020-018-0031-8)
Supplement: Supplementary file 2 — Top interactions from W-Z case-only test in confirmation set. Description: SNP1 and SNP2 are the two corresponding SNP candidates of a pair from the case-only population belonging to chromosomes denote by Chr1 and Chr2; gene1 and gene2 are the corresponding genes annotated to SNP1 and SNP2, respectively. W-Z P value is Wellek Ziegler case-only test p-value; BP is base pair. (DOCX 21 kb) [file 10020_2018_31_MOESM2_ESM.docx]

**Additional file 2.** Top interactions from W-Z case-only test in confirmation set. SNP1 and SNP2 are the two corresponding SNP candidates of a pair from the case-only population belonging to chromosomes denote by Chr1 and Chr2; gene1 and gene2 are the corresponding genes annotated to SNP1 and SNP2, respectively. W-Z P value is Wellek Ziegler case-only test p-value; BP is base pair.

|  |  |  |  |  |  |  |  |  |
| --- | --- | --- | --- | --- | --- | --- | --- | --- |
| **Gene1** | **Chr1** | **SNP1** | **BP1** | **Gene2** | **Chr2** | **SNP2** | **BP2** | **W-Z P value** |
| A2BP1 | 16 | rs4433825 | 7378192 | PLCB1 | 20 | rs2295179 | 8678446 | 3.35E-24 |
| LOC644624 | 4 | rs12509315 | 124592367 | RUNX1 | 21 | rs2242901 | 36456189 | 7.25E-20 |
| CNTNAP2 | 7 | rs1496547 | 146923861 | DDHD1 | 14 | rs1959843 | 53859798 | 3.43E-19 |
| RP1-21O18.1 | 1 | rs4543785 | 15089767 | LOC728721 | 3 | rs6796254 | 14401563 | 4.25E-19 |
| MTF1 | 1 | rs12743834 | 38318929 | NOVA1 | 14 | rs751401 | 26828666 | 1.71E-18 |
| KLHL29 | 2 | rs1368080 | 23711427 | NULL | 9 | rs12464177 | 133627136 | 3.40E-17 |
| KCNAB2 | 1 | rs546526 | 6070870 | hCG 2025798 | 4 | rs6836888 | 182285422 | 3.83E-17 |
| CMYA5 | 5 | rs259103 | 79094685 | ASTN2 | 9 | rs7875944 | 120162380 | 6.17E-17 |
| BUB3 | 10 | rs6599673 | 125008718 | DSEL | 18 | rs12966710 | 65286267 | 8.16E-17 |
| HSP90AB2P | 4 | rs3213705 | 13297604 | LOC728727 | 6 | rs17082113 | 120639122 | 9.68E-17 |
| RAPGEF2 | 4 | rs3846243 | 161292814 | CSMD1 | 8 | rs4875703 | 3224561 | 1.23E-16 |
| HSP90AB2P | 4 | rs3213705 | 13297604 | LOC728727 | 6 | rs17082113 | 120639122 | 2.16E-16 |
| GRM4 | 6 | rs2229901 | 33990447 | NULL | 6 | rs7175323 | 87351138 | 2.21E-16 |
| LOC391642 | 4 | rs6841769 | 27905536 | FAM84B | 8 | rs4557669 | 127306602 | 4.99E-16 |
| SGCZ | 8 | rs1565919 | 15238678 | A2BP1 | 16 | rs7198717 | 7688529 | 1.90E-15 |
| SGCZ | 8 | rs6981363 | 14476252 | A2BP1 | 16 | rs1476964 | 6624363 | 1.90E-15 |
| SATB1 | 3 | rs6550850 | 18507382 | GALNT17 | 4 | rs1394860 | 172979002 | 3.48E-15 |
| ERBB4 | 2 | rs2033643 | 212221870 | ODZ2 | 5 | rs4976582 | 167612389 | 4.93E-15 |
| LOC344371 | 2 | rs4670331 | 34410240 | COX18 | 4 | rs3762883 | 73930552 | 5.20E-15 |
| ERC2 | 3 | rs1795648 | 55571760 | SOX2OT | 3 | rs9845058 | 181342415 | 5.29E-15 |
| TTL | 2 | rs6718489 | 113280880 | PTPRD | 9 | rs6477256 | 8065584 | 7.40E-15 |
