# Supplementary material for: Enrichment of B cell receptor signaling and epidermal growth factor receptor pathways in monoclonal gammopathy of undetermined significance: a genome-wide genetic interaction study
Source: Mol Med. 2018 Jun 11;24:30. doi: 10.1186/s10020-018-0031-8 (PMC6016882; doi:10.1186/s10020-018-0031-8)
Supplement: Supplementary file 3 — Top interactions from W-Z interaction test on the replication set. Description: SNP1 and SNP2 are the two SNP candidates of a pair from the replication set population belonging to chromosomes denote by Chr1 and Chr2; gene1 and gene2 are the corresponding genes annotated to SNP1 and SNP2, respectively. W-Z P value is Wellek Ziegler case-control test p-value; BP is base pair. (DOCX 22 kb) [file 10020_2018_31_MOESM3_ESM.docx]

**Additional file 3.** Top interactions from W-Z interaction test on the replication set. SNP1 and SNP2 are the two SNP candidates of a pair from the replication set population belonging to chromosomes denote by Chr1 and Chr2; gene1 and gene2 are the corresponding genes annotated to SNP1 and SNP2, respectively. W-Z P value is Wellek Ziegler case-control test p-value; BP is base pair.

| **gene1** | **Chr1** | **SNP1** | **BP1** | **gene2** | **Chr2** | **SNP2** | **BP2** | **W-Z P value** |
| --- | --- | --- | --- | --- | --- | --- | --- | --- |
| FBXL17 | 5 | rs1799011 | 107325759 | MAGI2 | 7 | rs967489 | 78529329 | 2.59E-36 |
| LOC728394 | 4 | rs11090644 | 92431729 | FBLN1 | 22 | rs7677659 | 45982997 | 1.52E-35 |
| NELL1 | 11 | rs11640925 | 21308199 | A2BP1 | 16 | rs7127622 | 8156523 | 1.73E-35 |
| DTNBP1 | 6 | rs10266202 | 15625808 | NXPH1 | 7 | rs2743868 | 8957041 | 1.13E-34 |
| NULL | 4 | rs8181443 | 8541869 | RAB11FIP2 | 10 | rs6447879 | 119670998 | 3.07E-32 |
| IRX1 | 5 | rs12051446 | 3845683 | A2BP1 | 16 | rs9687393 | 7409031 | 5.21E-32 |
| PLXDC2 | 10 | rs2015847 | 20405138 | CDRT4 | 17 | rs2461941 | 15318855 | 7.52E-30 |
| ERBB4 | 2 | rs2144066 | 212974828 | DIO3OS | 14 | rs17416172 | 101938855 | 4.42E-29 |
| LOC646538 | 1 | rs7159563 | 81177525 | LOC730105 | 14 | rs841666 | 82783075 | 2.93E-28 |
| TSNARE1 | 8 | rs10904319 | 143233312 | LOC338588 | 10 | rs10110636 | 4741842 | 3.03E-28 |
| SOX11 | 2 | rs214742 | 5361397 | TMEM135 | 11 | rs10181393 | 86985581 | 4.05E-28 |
| LOC646538 | 1 | rs12588076 | 81177525 | LOC730105 | 14 | rs841666 | 82746477 | 1.42E-27 |
| TCERG1L | 10 | rs8045250 | 132963432 | A2BP1 | 16 | rs4751335 | 6879583 | 1.62E-27 |
| LOC344371 | 2 | rs2502294 | 34575895 | RCADH5 | 6 | rs7577875 | 67792729 | 1.69E-27 |
| FHIT | 3 | rs909876 | 61177577 | DHX35 | 20 | rs7617424 | 38025235 | 1.78E-27 |
| COL9A1 | 6 | rs509333 | 71064295 | MN1 | 22 | rs7772055 | 27640747 | 2.71E-27 |
| NKAIN2 | 6 | rs10759037 | 124200765 | PTPRD | 9 | rs9388287 | 9064330 | 4.52E-27 |
| NSUN2 | 5 | rs11070218 | 6599222 | LOC644779 | 15 | rs6876835 | 39823058 | 8.56E-27 |
| RAP2B | 3 | rs10267303 | 152994772 | AUTS2 | 7 | rs7355869 | 70082913 | 9.61E-27 |
| UHRF2 | 9 | rs7983829 | 6459274 | MYO16 | 13 | rs524888 | 109736676 | 1.25E-26 |
| ROBO2 | 3 | rs6575656 | 77720924 | C14orf177 | 14 | rs9883373 | 98761422 | 1.49E-26 |
| PTPRD | 9 | rs4755435 | 8227101 | LDLRAD3 | 11 | rs10976860 | 36136580 | 1.94E-26 |
| RIMS1 | 6 | rs4820127 | 72908366 | LOC730062 | 22 | rs1852702 | 34395171 | 2.74E-26 |
| LOC390419 | 13 | rs3859840 | 91448197 | ISX | 22 | rs2152310 | 35359702 | 3.04E-26 |
| LOC388474 | 18 | rs134794 | 36232589 | MN1 | 22 | rs1540018 | 27668370 | 3.05E-26 |
| HTR1B | 6 | rs12193281 | 78298165 | SNAP91 | 6 | rs2252216 | 84325184 | 5.75E-26 |
