# Supplementary material for: Enrichment of B cell receptor signaling and epidermal growth factor receptor pathways in monoclonal gammopathy of undetermined significance: a genome-wide genetic interaction study
Source: Mol Med. 2018 Jun 11;24:30. doi: 10.1186/s10020-018-0031-8 (PMC6016882; doi:10.1186/s10020-018-0031-8)
Supplement: Supplementary file 4 — Overlapped interactions from INTERSNP and CASSI W-Z interaction tests on discovery set. Description: SNP1 and SNP2 are the two SNP candidates of a pair from the discovery set population belonging to chromosomes denote by Chr1 and Chr2; gene1 and gene2 are the corresponding genes annotated to SNP1(s) and SNP2(s), respectively. W-Z P value is Wellek Ziegler case-control test p-value from CASSI and INTERSNP P value is full log-linear test p-value from INTERSNP; BP is base pair. IN: INTERSNP; CAS: CASSI. (DOCX 22 kb) [file 10020_2018_31_MOESM4_ESM.docx]

**Additional file 4.** Overlapped interactions from INTERSNP and CASSI W-Z interaction tests on discovery set. SNP1 and SNP2 are the two SNP candidates of a pair from the discovery set population belonging to chromosomes denote by Chr1 and Chr2; gene1 and gene2 are the corresponding genes annotated to SNP1(s) and SNP2(s), respectively. W-Z P value is Wellek Ziegler case-control test p-value from CASSI and INTERSNP P value is full log-linear test p-value from INTERSNP; BP is base pair. IN: INTERSNP; CAS: CASSI.

| **gene1** | **Chr1** | **SNP1 (IN)** | **gene2** | **Chr2** | **SNP2 (IN)** | **INTERSNP**  **P value** | **SNP1 (CAS)** | **SNP2 (CAS)** | **W-Z P value** |
| --- | --- | --- | --- | --- | --- | --- | --- | --- | --- |
| AJAP1 | 1 | rs1878052 | LRP1B | 2 | rs6429882 | 8.16E-06 | rs9286971 | rs12476507 | 7.90E-07 |
| CRIM1 | 2 | rs3770827 | GPC5 | 13 | rs9516137 | 3.14E-06 | rs1167459 | rs9523782 | 2.53E-07 |
| CSMD1 | 8 | rs4517143 | CSMD3 | 8 | rs1017829 | 1.07E-06 | rs6558973 | rs373880 | 9.75E-07 |
| CSMD1 | 8 | rs4517143 | CSMD3 | 8 | rs1017829 | 1.07E-06 | rs2623657 | rs13257287 | 3.22E-07 |
| CSMD1 | 8 | rs4517143 | CSMD3 | 8 | rs1017829 | 1.07E-06 | rs6558973 | rs370168 | 9.64E-07 |
| CSMD1 | 8 | rs4517143 | CSMD3 | 8 | rs1017829 | 1.07E-06 | rs6558973 | rs13261160 | 9.62E-07 |
| CSMD1 | 8 | rs4517143 | CSMD3 | 8 | rs1017829 | 1.07E-06 | rs2623657 | rs13257287 | 3.22E-07 |
| CSMD1 | 8 | rs4517143 | CSMD3 | 8 | rs1017829 | 1.07E-06 | rs6558973 | rs231317 | 9.63E-07 |
| CSMD1 | 8 | rs17066034 | FRMD4A | 10 | rs7082219 | 3.76E-06 | rs2977715 | rs11258541 | 4.40E-07 |
| CSMD1 | 8 | rs17066034 | FRMD4A | 10 | rs7082219 | 3.76E-06 | rs2978315 | rs789680 | 5.84E-08 |
| CSMD1 | 8 | rs17066034 | FRMD4A | 10 | rs7082219 | 3.76E-06 | rs2977715 | rs11258541 | 4.40E-07 |
| CSMD1 | 8 | rs17066034 | FRMD4A | 10 | rs7082219 | 3.76E-06 | rs2124153 | rs789680 | 1.96E-07 |
| GALNT14 | 2 | rs1559497 | LOC387939 | 13 | rs9546709 | 2.43E-06 | rs2161834 | rs9575620 | 8.94E-07 |
| GRK5 | 10 | rs4752292 | PLCB1 | 20 | rs708925 | 4.54E-06 | rs7093673 | rs6086518 | 3.03E-07 |
| GRM7 | 3 | rs7638535 | LOC391273 | 21 | rs7278309 | 6.67E-06 | rs332936 | rs13048376 | 5.30E-07 |
| LOC729360 | 5 | rs307177 | CSMD1 | 8 | rs2623625 | 3.70E-06 | rs306254 | rs12546362 | 1.35E-07 |
| LOC730057 | 3 | rs704457 | CSMD1 | 8 | rs918150 | 7.14E-06 | rs793291 | rs2128219 | 8.90E-08 |
| LRP1B | 2 | rs1370328 | NULL | 2 | rs281583 | 8.51E-06 | rs7607195 | rs4860411 | 7.13E-07 |
| LRP1B | 2 | rs16846362 | NULL | 16 | rs4888670 | 8.12E-06 | rs7607195 | rs4860411 | 7.13E-07 |
| NULL | 16 | rs7203274 | LOC255130 | 4 | rs12648067 | 9.83E-08 | rs4594588 | rs7679186 | 6.16E-07 |
| NULL | 4 | rs6447879 | LOC647317 | 13 | rs9602023 | 8.34E-06 | rs6470556 | rs9318893 | 1.58E-08 |
| NXPH1 | 7 | rs17153994 | CLYBL | 13 | rs7982361 | 3.07E-06 | rs12536728 | rs1555589 | 8.24E-07 |
| PTPRD | 9 | rs7032880 | FTHL7 | 13 | rs9510052 | 4.45E-06 | rs16929713 | rs465174 | 3.12E-07 |
| ST6GALNAC3 | 1 | rs12075814 | HACE1 | 6 | rs17065282 | 1.67E-06 | rs2893416 | rs6571174 | 6.94E-07 |
| TCP11L1 | 11 | rs1064005 | MACROD2 | 20 | rs6043648 | 1.00E-06 | rs2024967 | rs6043561 | 6.86E-07 |
| TCP11L1 | 11 | rs1064005 | MACROD2 | 20 | rs6043648 | 1.00E-06 | rs2024967 | rs6043565 | 9.93E-07 |
