# Supplementary material for: Enrichment of B cell receptor signaling and epidermal growth factor receptor pathways in monoclonal gammopathy of undetermined significance: a genome-wide genetic interaction study
Source: Mol Med. 2018 Jun 11;24:30. doi: 10.1186/s10020-018-0031-8 (PMC6016882; doi:10.1186/s10020-018-0031-8)
Supplement: Supplementary file 5 — Gene set enrichment analysis in genetic network with STRING. Description: Based on the indexing nodes and the additional predicted first order interacting m=nodes, STRING performs enrichment analysis on several molecular, biological, cellular process related pathway analysis with Gene Onltology (GO) and KEGG database. All tests are performed with guilt by association assumption and P values are corrected for multiple testing. A protein-protein enrichment index is reported with analysis depicting level of confidence in the detected enriched processes which is reported to be 0.0039 (significant at 5% level). (DOCX 21 kb) [file 10020_2018_31_MOESM5_ESM.docx]

**Additional file 5.** Gene set enrichment analysis in genetic network with STRING. Based on the indexing nodes and the additional predicted first order interacting nodes, STRING performs enrichment analysis on several molecular, biological, cellular process related pathway analysis with Gene Onltology (GO) and KEGG database. All tests are performed with guilt by association assumption and *P* values are corrected for multiple testing. A protein-protein enrichment index is reported with analysis depicting level of confidence in the detected enriched processes which is reported to be 0.0039 (significant at 5% level)

| **pathway description** | **observed gene count** | **P value** |  | **pathway description** | **observed gene count** | **P value** |
| --- | --- | --- | --- | --- | --- | --- |
| ErbB signaling pathway | 5 | 7.09E-05 |  | Neurotrophin signaling pathway | 3 | 1.32E-02 |
| B cell receptor signaling pathway | 3 | 5.32E-03 |  | Thyroid cancer | 2 | 1.32E-02 |
| Fc epsilon RI signaling pathway | 3 | 5.32E-03 |  | FoxO signaling pathway | 3 | 1.38E-02 |
| Prolactin signaling pathway | 3 | 5.32E-03 |  | Natural killer cell mediated cytotoxicity | 3 | 1.38E-02 |
| MicroRNAs in cancer | 4 | 5.32E-03 |  | Circadian rhythm | 2 | 1.38E-02 |
| Renal cell carcinoma | 3 | 5.32E-03 |  | Insulin signaling pathway | 3 | 1.62E-02 |
| Endometrial cancer | 3 | 5.32E-03 |  | Parkinson s disease | 3 | 1.73E-02 |
| Glioma | 3 | 5.32E-03 |  | Prion diseases | 2 | 1.73E-02 |
| Chronic myeloid leukemia | 3 | 5.32E-03 |  | Hepatitis B | 3 | 1.73E-02 |
| Acute myeloid leukemia | 3 | 5.32E-03 |  | Bladder cancer | 2 | 1.76E-02 |
| Non-small cell lung cancer | 3 | 5.32E-03 |  | PI3K-Akt signaling pathway | 4 | 2.22E-02 |
| Gap junction | 3 | 8.29E-03 |  | Chemokine signaling pathway | 3 | 3.04E-02 |
| GnRH signaling pathway | 3 | 8.29E-03 |  | Long-term depression | 2 | 3.87E-02 |
| Prostate cancer | 3 | 8.29E-03 |  | VEGF signaling pathway | 2 | 3.88E-02 |
| Proteoglycans in cancer | 4 | 8.66E-03 |  | Focal adhesion | 3 | 3.94E-02 |
| Estrogen signaling pathway | 3 | 8.82E-03 |  | Long-term potentiation | 2 | 4.15E-02 |
| Dorso-ventral axis formation | 2 | 9.67E-03 |  | Ras signaling pathway | 3 | 4.65E-02 |
| T cell receptor signaling pathway | 3 | 9.67E-03 |  | Melanoma | 2 | 4.68E-02 |
