# Supplementary material for: Enrichment of B cell receptor signaling and epidermal growth factor receptor pathways in monoclonal gammopathy of undetermined significance: a genome-wide genetic interaction study
Source: Mol Med. 2018 Jun 11;24:30. doi: 10.1186/s10020-018-0031-8 (PMC6016882; doi:10.1186/s10020-018-0031-8)
Supplement: Supplementary file 6 — PASCAL gene set enrichment analysis results at 1% level of significance. (DOCX 20 kb) [file 10020_2018_31_MOESM6_ESM.docx]

**Additional file 6.** PASCAL gene set enrichment analysis results at 1% level of significance

| **Database** | **Pathway** | **P value** |
| --- | --- | --- |
| REACTOME | METABOLISM OF POLYAMINES | 3.77E-05 |
| REACTOME | THROMBIN SIGNALLING THROUGH PROTEINASE ACTIVATED RECEPTORS PARS | 6.17E-04 |
| REACTOME | GPCR DOWNSTREAM SIGNALING | 8.98E-04 |
| REACTOME | THROMBOXANE SIGNALLING THROUGH TP RECEPTOR | 9.35E-04 |
| REACTOME | G ALPHA Z SIGNALLING EVENTS | 1.24E-03 |
| REACTOME | KERATAN SULFATE BIOSYNTHESIS | 1.31E-03 |
| REACTOME | KERATAN SULFATE KERATIN METABOLISM | 2.38E-03 |
| REACTOME | SIGNAL AMPLIFICATION | 2.62E-03 |
| REACTOME | ADP SIGNALLING THROUGH P2RY1 | 3.44E-03 |
| REACTOME | G ALPHA I SIGNALLING EVENTS | 4.07E-03 |
| REACTOME | AMINE LIGAND BINDING RECEPTORS | 4.54E-03 |
| BIOCARTA | TCRA PATHWAY | 4.76E-03 |
| REACTOME | AQUAPORIN MEDIATED TRANSPORT | 5.40E-03 |
| REACTOME | PROSTACYCLIN SIGNALLING THROUGH PROSTACYCLIN RECEPTOR | 5.87E-03 |
| KEGG | PARKINSONS DISEASE | 8.18E-03 |
| REACTOME | G ALPHA Q SIGNALLING EVENTS | 8.43E-03 |
| KEGG | GLYCOSAMINOGLYCAN BIOSYNTHESIS KERATAN SULFATE | 8.45E-03 |
| REACTOME | GLUCAGON TYPE LIGAND RECEPTORS | 9.74E-03 |
| REACTOME | GPCR LIGAND BINDING | 9.85E-03 |
