# Supplementary material for: Enrichment of B cell receptor signaling and epidermal growth factor receptor pathways in monoclonal gammopathy of undetermined significance: a genome-wide genetic interaction study
Source: Mol Med. 2018 Jun 11;24:30. doi: 10.1186/s10020-018-0031-8 (PMC6016882; doi:10.1186/s10020-018-0031-8)
Supplement: Supplementary file 7 — MAGENTA gene set enrichment analysis results at 1% level of significance. (DOCX 20 kb) [file 10020_2018_31_MOESM7_ESM.docx]

**Additional file 7.**  MAGENTA gene set enrichment analysis results at 1% level of significance.

| **Data Base** | **Pathway** | **P Value** |
| --- | --- | --- |
| Ingenuity | T Cell Receptor Signaling | 1.10E-03 |
| REACTOME | CD28 dependent vav1 pathway | 1.30E-03 |
| PANTHER BIOLOGICAL PROCESS | Blood clotting | 1.80E-03 |
| PANTHER BIOLOGICAL PROCESS | Purine metabolism | 2.00E-03 |
| GOTERM | Nucleotide binding | 2.50E-03 |
| GOTERM | Cilium assembly | 3.20E-03 |
| REACTOME | EGFR downregulation | 3.20E-03 |
| REACTOME | Integrin alphaiibbeta3 signaling | 3.50E-03 |
| GOTERM | Odontogenesis | 4.10E-03 |
| GOTERM | Small GTPase mediated signal transduction | 4.60E-03 |
| GOTERM | Regulation of cell shape | 4.70E-03 |
| KEGG | KEGG allograft rejection | 5.10E-03 |
| GOTERM | Lipopolysaccharide binding | 5.40E-03 |
| REACTOME | Platelet aggregation plug formation | 5.50E-03 |
| GOTERM | Intracellular protein transport | 6.30E-03 |
| PANTHER MOLECULAR FUNCTION | Interleukin | 7.20E-03 |
| GOTERM | Positive regulation of interleukin-8 production | 7.50E-03 |
| BIOCARTA | ASBcell pathway | 8.30E-03 |
| PANTHER MOLECULAR FUNCTION | Non-receptor tyrosine protein kinase | 8.40E-03 |
| BIOCARTA | DC pathway | 8.40E-03 |
| PANTHER BIOLOGICAL PROCESS | Cell cycle | 9.20E-03 |
| Panther | Cytoskeletal regulation by Rho GTPase | 1.00E-02 |
