# Supplementary material for: Enrichment of B cell receptor signaling and epidermal growth factor receptor pathways in monoclonal gammopathy of undetermined significance: a genome-wide genetic interaction study
Source: Mol Med. 2018 Jun 11;24:30. doi: 10.1186/s10020-018-0031-8 (PMC6016882; doi:10.1186/s10020-018-0031-8)
Supplement: Supplementary file 8 — All detected pathways mutually discovered in both MAGENTA and PASCAL at a 5% level of combined significance. (DOCX 21 kb) [file 10020_2018_31_MOESM8_ESM.docx]

|  |  | **PASCAL** | **MAGENTA** |  |
| --- | --- | --- | --- | --- |
| Database | Pathway | P value | P value | Combined P value |
| REACTOME | CD28 dependent vav1 pathway | 4.55E-02 | 1.30E-03 | 5.92E-05 |
| REACTOME | Platelet aggregation plug formation | 4.40E-02 | 5.50E-03 | 2.42E-04 |
| KEGG | Glycosaminoglycan biosynthesis keratan sulfate | 8.45E-03 | 3.65E-02 | 3.08E-04 |
| KEGG | Allograft rejection | 8.38E-02 | 5.10E-03 | 4.27E-04 |
| REACTOME | G protein activation | 1.40E-02 | 3.74E-02 | 5.22E-04 |
| KEGG | Type 1 diabetes mellitus | 4.48E-02 | 1.94E-02 | 8.69E-04 |
| BIOCARTA | Asbcell pathway | 1.10E-01 | 8.30E-03 | 9.15E-04 |
| KEGG | Autoimmune thyroid disease | 4.32E-02 | 2.24E-02 | 9.68E-04 |
| REACTOME | P130cas linkage to mapk signaling for integrins | 3.60E-02 | 3.23E-02 | 1.16E-03 |
| REACTOME | Integrin cell surface interactions | 1.61E-01 | 1.11E-02 | 1.78E-03 |
| REACTOME | EGFR downregulation | 7.77E-01 | 3.20E-03 | 2.49E-03 |
| BIOCARTA | DC pathway | 3.50E-01 | 8.40E-03 | 2.94E-03 |
| BIOCARTA | Integrin pathway | 8.65E-02 | 3.62E-02 | 3.13E-03 |
| KEGG | Dorso ventral axis formation | 1.52E-01 | 2.20E-02 | 3.34E-03 |
| REACTOME | mRNA 3 end processing | 2.18E-01 | 1.90E-02 | 4.14E-03 |
| REACTOME | Toll receptor cascades | 3.90E-01 | 1.18E-02 | 4.60E-03 |
| BIOCARTA | Th1th2 pathway | 1.20E-01 | 4.40E-02 | 5.26E-03 |
| BIOCARTA | Ach pathway | 1.56E-01 | 4.03E-02 | 6.28E-03 |
| BIOCARTA | CTLA4 pathway | 2.03E-01 | 4.25E-02 | 8.64E-03 |
| REACTOME | Sema3a pak dependent axon repulsion | 2.96E-01 | 2.93E-02 | 8.66E-03 |
| REACTOME | MAPK targets nuclear events mediated by map kinases | 1.98E-01 | 4.52E-02 | 8.97E-03 |
| REACTOME | FGFR ligand binding and activation | 2.95E-01 | 3.10E-02 | 9.14E-03 |
| REACTOME | Signaling by robo receptor | 4.78E-01 | 1.92E-02 | 9.19E-03 |
| REACTOME | CD28 co stimulation | 1.91E-01 | 4.83E-02 | 9.25E-03 |
| KEGG | Intestinal immune network for iga production | 5.99E-01 | 2.14E-02 | 1.28E-02 |
| BIOCARTA | Cytokine pathway | 3.29E-01 | 4.55E-02 | 1.50E-02 |
| REACTOME | CDT1 association with the cdc6 orc origin complex | 6.42E-01 | 3.38E-02 | 2.17E-02 |
| BIOCARTA | MAPK pathway | 4.66E-01 | 4.67E-02 | 2.17E-02 |

**Additional file 8.** All detected pathways mutually discovered in both MAGENTA and PASCAL at a 5% level of combined significance.
