# Supplementary material for: Enrichment of B cell receptor signaling and epidermal growth factor receptor pathways in monoclonal gammopathy of undetermined significance: a genome-wide genetic interaction study
Source: Mol Med. 2018 Jun 11;24:30. doi: 10.1186/s10020-018-0031-8 (PMC6016882; doi:10.1186/s10020-018-0031-8)
Supplement: Supplementary file 9 — DEPICT gene set enrichment analysis results at Bonferroni corrected genome wide significance level. (DOCX 18 kb) [file 10020_2018_31_MOESM9_ESM.docx]

| **Original gene set ID** | **Original gene set description** | **Nominal P value** | **FDR < 5%** |
| --- | --- | --- | --- |
| ENSEMBLE | BCL2A1 subnetwork | 7.09E-11 | Yes |
| GOTERM | T cell activation | 1.87E-09 | Yes |
| MP:0006414 | Decreased T cell apoptosis | 2.61E-09 | Yes |
| MP:0002416 | Abnormal proerythroblast morphology | 9.89E-09 | Yes |

**Additional file 9.** DEPICT gene set enrichment analysis results at Bonferroni corrected genome wide significance level.
