# Supplementary material for: Enrichment of B cell receptor signaling and epidermal growth factor receptor pathways in monoclonal gammopathy of undetermined significance: a genome-wide genetic interaction study
Source: Mol Med. 2018 Jun 11;24:30. doi: 10.1186/s10020-018-0031-8 (PMC6016882; doi:10.1186/s10020-018-0031-8)
Supplement: Supplementary file 10 — Summary of Illumina bead chips used for genotyping different batches of cases and controls. (DOCX 19 kb) [file 10020_2018_31_MOESM10_ESM.docx]

|  | **Genotyping chip** | **Number of SNPs** | **No. of cases** | **No. of controls** |
| --- | --- | --- | --- | --- |
| **Chip1** | Illumina HumanCoreExome-12v1-1 | 542,585 | ^c^ 15 | ^c^ 350 |
| **Chip2** | Illumina HumanCoreExome-12v1-0 | 538,448 | ^c^ 82 | ^c^ 1409 |
| **Chip3** | Illumina Human660W-Quad v1 | 657,366 | ^c^ 119 | ^c^ 14 |
| **Chip4** | Illumina HumanOmni-Quad V.1 | 1,140,419 | ^c^ 45 | ^c^ 766 |
| **Chip5** | Illumina HumanOmniExpress 12v1.0 | 730,525 | ^b^ 82 | ^a^ 1303 |
| **Chip6** | Illumina HumanOmniExpress-12v1.1 | 730,725 | ^a^ 243 | N/A |

**Additional file 10.** Summary of Illumina bead chips used for genotyping different batches of cases and controls.

^a^ Discovery set cases and controls

^b^ Follow up set cases

^c^ Replication set cases and controls
