# Supplementary material for: Enrichment of B cell receptor signaling and epidermal growth factor receptor pathways in monoclonal gammopathy of undetermined significance: a genome-wide genetic interaction study
Source: Mol Med. 2018 Jun 11;24:30. doi: 10.1186/s10020-018-0031-8 (PMC6016882; doi:10.1186/s10020-018-0031-8)
Supplement: Supplementary file 11 — Number of overlaps in number of SNPs prior quality control between different chips used in genotyping. Chip numbers are defined in Additional file 10. (DOCX 19 kb) [file 10020_2018_31_MOESM11_ESM.docx]

|  | **Chip1** | **Chip2** | **Chip3** | **Chip4** | **Chip5** | **Chip6** |
| --- | --- | --- | --- | --- | --- | --- |
| **Chip1** | 542585 | 535478 | 128261 | 244172 | 252942 | 205700 |
| **Chip2** |  | 538448 | 128337 | 244385 | 253159 | 205723 |
| **Chip3** |  |  | 657366 | 392615 | 324520 | 266040 |
| **Chip4** |  |  |  | 1140419 | 706093 | 534858 |
| **Chip5** |  |  |  |  | 730525 | 534604 |
| **Chip6** |  |  |  |  |  | 730725 |

**Additional file 11.** Number of overlaps in number of SNPs prior quality control between different chips used in genotyping. Chip numbers are defined in **Additional file 10**.
